# Human decision-making biases in the moral dilemmas of autonomous vehicles

**DOI:** 10.1038/s41598-019-49411-7

**Published:** 2019-09-11

**Authors:** Darius-Aurel Frank, Polymeros Chrysochou, Panagiotis Mitkidis, Dan Ariely

**Affiliations:** 1 Department of Management, Aarhus University, Aarhus, Denmark; 2 Ehrenberg-Bass Institute for Marketing Science, School of Marketing, University of South Australia, South Australia, Australia; 3 Center for Advanced Hindsight, Duke University, Durham, United States

**Keywords:** Social behaviour, Human behaviour

## Abstract

The development of artificial intelligence has led researchers to study the ethical principles that should guide machine behavior. The challenge in building machine morality based on people’s moral decisions, however, is accounting for the biases in human moral decision-making. In seven studies, this paper investigates how people’s personal perspectives and decision-making modes affect their decisions in the moral dilemmas faced by autonomous vehicles. Moreover, it determines the variations in people’s moral decisions that can be attributed to the situational factors of the dilemmas. The reported studies demonstrate that people’s moral decisions, regardless of the presented dilemma, are biased by their decision-making mode and personal perspective. Under intuitive moral decisions, participants shift more towards a deontological doctrine by sacrificing the passenger instead of the pedestrian. In addition, once the personal perspective is made salient participants preserve the lives of that perspective, i.e. the passenger shifts towards sacrificing the pedestrian, and vice versa. These biases in people’s moral decisions underline the social challenge in the design of a universal moral code for autonomous vehicles. We discuss the implications of our findings and provide directions for future research.

## Introduction

Autonomous vehicles are at the forefront of the development of artificial intelligence and are designed to operate without any human intervention^1^. It is expected that they will revolutionize public and private transportation, with the prospect of saving lives, reducing congestion, enhancing mobility, and improving overall productivity^2–6^. The future of autonomous vehicles, however, is disputed due to the ethical and psychological concerns about their behavior in critical, non-routine traffic situations that potentially involve fatalities^7–9^. The challenge in training artificial intelligence morality is meeting societal expectations about the ethical principles that should guide machine behavior^10^. An unresolved question is how an autonomous vehicle should be trained to act when – regardless of its actions – the outcome of a critical incident would lead to fatality^8,11^.

To address this challenge, researchers set out to explore the moral dilemmas faced by autonomous vehicles in order to develop a universally accepted moral code that could guide the machines’ behavior^10,12,13^. The largest project, the Moral Machine experiment, an online experimental platform designed to explore the moral dilemmas faced by autonomous vehicles, has managed to gather data on millions of humans’ moral decisions^10^. This data was consecutively used to train machine-learning algorithms^14^, such as those implemented in autonomous vehicles. Developing moral guidelines for artificial intelligence driven technologies based on people’s moral decisions, however, risks incorporating human predispositions in moral decision-making^15^. The most prevalent conditions that were shown to interfere with moral judgements are cognitive load^16^ and emotional engagement^15^.

The inherent problem of peoples’ preferences in moral dilemmas, as discussed by Bonnefon and colleagues, is that people seem to favor a utilitarian moral doctrine that minimizes the total casualties in potentially fatal accidents, but they simultaneously report preferring an autonomous vehicle that is preprogrammed to protect themselves and their families over the lives of others^14^. These findings illustrate that moral decisions could be a matter of personal perspective: When people think about the outcomes of the dilemmas for the greater good of society, they appear to employ a utilitarian moral doctrine; however, when they consider themselves and their loved ones, they shift towards a deontological moral doctrine that rejects the idea of sacrificing the passengers in their vehicle^17^. As a consequence, moral codes derived from human decisions could reflect biased moral preferences.

Our research aims to investigate whether the abovementioned mechanisms can help explain the duality in people’s moral decisions. In seven studies, we replicate moral dilemmas used in the Moral Machine experiment. First, we quantify the influence of personal perspective (passenger versus pedestrian) and decision-making mode (intuitive versus deliberate) on people’s decisions in moral dilemmas faced by autonomous vehicles. Second, we document the variations in moral preferences based on the situational factors of the dilemmas, including the number of passengers and pedestrians, presence of children among passengers and pedestrians, outcome of intervention, and lawfulness of pedestrians’ behavior. We discuss the implications of our findings for the development of universally accepted moral guidelines for autonomous vehicles and provide directions for future research.

## Biases in Moral Decision-Making

The dilemmas studied in research on the moral programming of autonomous vehicles represent adaptions of the original trolley problem^18^. The trolley problem describes a thought experiment in which an individual, confronted with the critical situation of a trolley about to run over five people, must choose between the default and an alternative outcome that alters the path of the trolley and sacrifices its single driver to save the people on the tracks. This dilemma can be transferred to the case of autonomous vehicles because the only difference is that autonomous vehicles are programmed in advance to make the decision^8^.

### Decision-making modes

Research on the underlying mechanisms of human moral decision-making in variations of the trolley dilemma shows that two distinct decision-making modes can alter the outcomes of people’s decision processes significantly^16^. Extending on dual-process theory^19–22^, Greene *et al*. find that in a deliberate decision-making mode, people use more cognitive resources and make more utilitarian moral decisions^16^. On the other hand, in the alternative, intuitive decision-making mode, which is driven by emotions and easily accessible rules, people make more deontological moral decisions. Based on the availability of processing time, people shift between the two modes^16^. Experiments show that in the presence of time pressure, people are systematically biased towards using the intuitive decision-making mode resulting in more deontological moral decisions^15,23^. Accordingly, it can be expected that people in a deliberate decision mode prefer a utilitarian moral code for autonomous vehicles that maximizes the number of saved lives, yet while in an intuitive decision mode categorically decide to sacrifice the passengers of the vehicle. This is why the environment and circumstances under which people face moral dilemmas can heavily influence those decisions’ outcomes. While the largest differences are likely to be observed between decisions in real-world actions (i.e., driver on the road) and hypothetical scenarios (i.e., taking a survey at home), the influence of people’s decision-making mode also applies when researchers survey large segments of the population for the purpose of programming autonomous vehicles to make moral decisions in dilemmas.

### Personal perspective

Another bias, when it comes to moral decisions in the dilemmas that are faced by autonomous vehicles, is rooted in the psychological constraints of people’s beliefs and decisions. The underlying theory of bounded rationality postulates that people’s decisions are biased by the cognitive limitations of their minds^24,25^, resulting in being biased by the emotional proximity of the event or people in question^26^. In support of this theory, Greene^27^ studies the moral bias that is attributed to emotional proximity in the realm of moral decision-making and finds that impersonal moral dilemmas are more likely to trigger utilitarian moral decisions, whereas personal dilemmas tend to result in more deontological moral decisions. Other research links this self-preserving behavior to personal perspective^28^. This bias of personal perspective can further be seen in recent findings of research on the moral dilemmas in the use of autonomous vehicles, which observes shifting moral judgements when it comes to people’s moral decisions for others and consideration of themselves^7^.

## Overview of the Present Research

Our research reports seven studies on human moral decision-making in the moral dilemmas faced by autonomous vehicles. The independent samples combine more than 12,000 moral decisions from thousands of individuals across the US and Denmark. The studies are designed to replicate the Moral Machine experiment, an online experimental platform that explored moral preferences in visual illustrations of the moral dilemmas faced by autonomous vehicles^10^. This design allows us to validate and discuss the findings in light of previous research and facilitates our contribution to the relevant understanding and future research.

Studies 1 and 2 investigate the influence of perspective and decision-making mode in people’s moral decisions in the context of the most basic and simple autonomous vehicle dilemma. Studies 3 and 4 gradually increase the complexity of the dilemma and test additional hypotheses on the underlying moral doctrines (deontological versus utilitarian) that guide people’s moral decisions. Studies 5 and 6 experiment with the concepts of agency and social norms violations and their influence on people’s moral decisions. Finally, Study 7 combines the cited factors in a large, controlled lab experiment and, along with an internal meta-analysis of the six online studies, provides converging evidence on the findings and conclusions of this research.

## Study 1: Perspective and Decision-making Mode

Study 1 establishes the main effects of decision-making mode and personal perspective on people’s decisions in the context of a simplified dilemma in which an autonomous vehicle must sacrifice either an innocent pedestrian or its own passenger. The purpose of this study is to determine whether the personal perspective of a decision-maker leads to more selfish and self-preserving moral decisions, according to which people from the perspective of the pedestrian will favor the life of the pedestrian. Moreover, it determines the extent to which moral decisions are affected by people’s decision-making mode, contrasting between intuitive and deliberate, reflective moral decisions.

## Method

### Participants

Eight hundred and seven participants (46.0% females; age: *M* = 32.49 years, *SD* = 11.84) were recruited on Prolific Academic and compensated $0.18–$0.35 each. Only US residents of minimum 18 years of age who were fluent in English were eligible for this study. The majority of participants reported having a driver’s license (89.1%) and using cars frequently (*M* = 5.93, *SD* = 1.51; 7-point scale, “7” very often, “1” never).

### Stimuli

**Figure 1:**
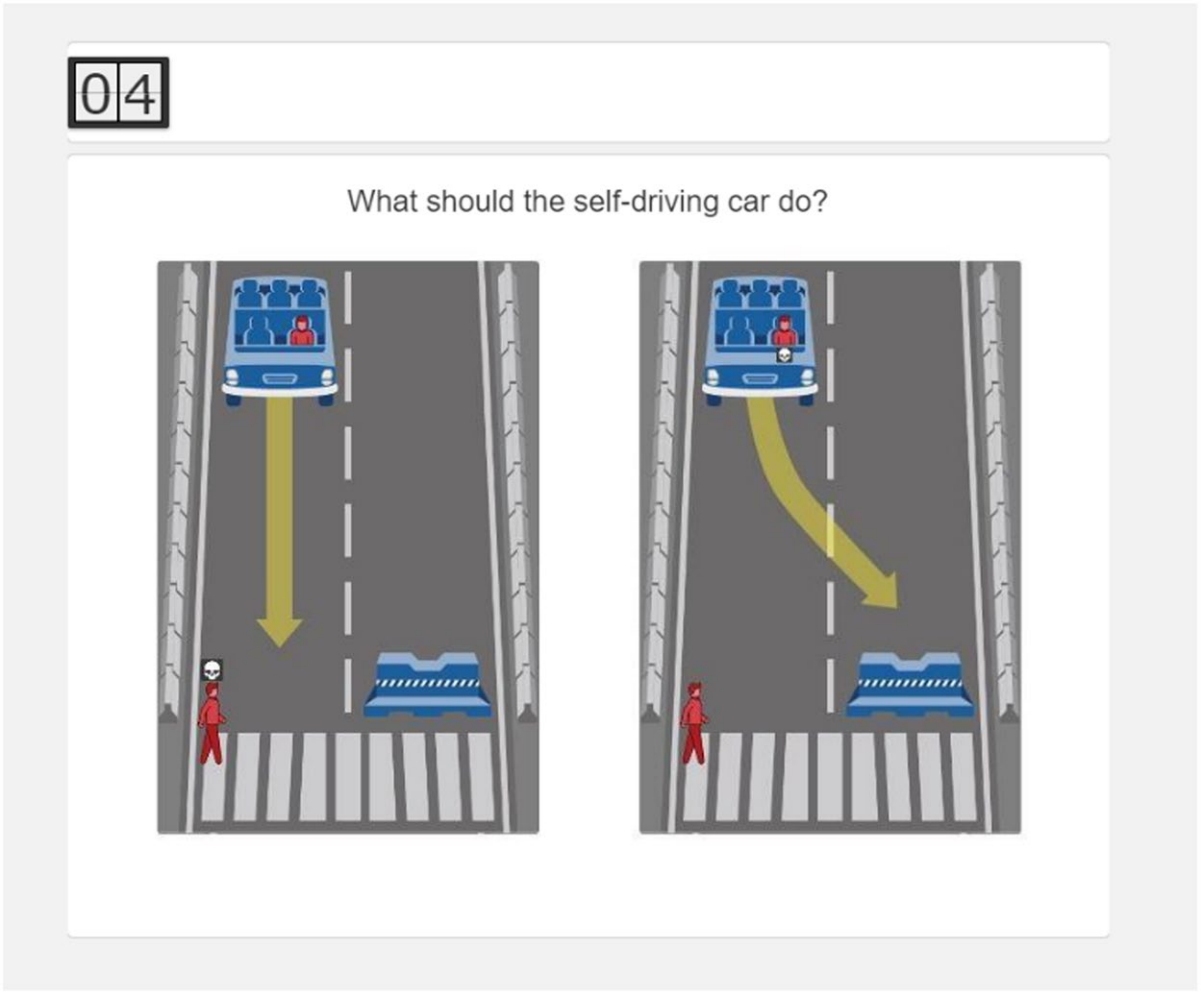
Moral decision task used in Study 1. Note: Own work. Image assets adapted from the Moral Machine (http://moralmachine.mit.edu/) by Scalable Cooperation and MIT Media Lab [CC BY 4.0 (https://creativecommons.org/licenses/by/4.0/)].

The stimuli used in this study and subsequent ones were adapted from the Moral Machine experiment (available at https://moralmachine.mit.edu/). The dilemma represents a modern variant of the original trolley dilemma^18^, in which an autonomous vehicle faces a critical incident with inevitably fatal consequences (see Supplementary materials for stimuli used in all studies). The decision-maker must choose between two possible outcomes: the autonomous vehicle either (a) stays on its original course, thereby killing one or more pedestrians crossing the street, or (b) swerves into the other lane, thereby killing one or more of its passengers. Figure 1 shows the simplified version of the studied dilemma, which consists of a single pedestrian and a single passenger and is presented to the decision-maker as two side-by-side illustrations of the possible, alternative outcomes. A timer in the top left corner indicates the remaining number of seconds to complete the task. Participants are instructed to select the outcome they believe is correct for an autonomous vehicle to be programmed to do.

### Design

Participants were randomly assigned to 3 perspectives (passenger, pedestrian, observer) x 2 decision-making modes (deliberate, intuitive) between-subject conditions. Perspectives consisted of two personal conditions (passenger, pedestrian) and a control condition (observer). In the personal perspectives, participants saw a visual stimulus of the target person with the instruction “Imagine that you are the [passenger of the car; pedestrian walking the street].” In the control condition, participants were only instructed to “Imagine […] you are observing the situation,” without visual aid.

Decision-making modes consisted of intuitive and deliberate conditions that were controlled for by manipulation of time pressure^15^. Time pressure was used to trigger intuitive decisions. In this condition, participants were instructed to respond in less than five seconds, a span of time that was pretested in a pilot study (*N* = 26; see Supplementary materials). In the deliberate decision-making condition, participants were instructed to respond within 30 seconds allowing them to make more deliberate, informed decisions. In both conditions, participants were presented with a visual timer that counted down the remaining seconds (see Fig. 1).

Due to a technical constraint in the survey software, participants were able to exceed the time limit of their respective condition. This limitation potentially affected participants who did not submit a decision before the timer ran out. We control for this limitation by excluding participants who responded (a) two or more seconds slower than the given time limit in the intuitive decision condition (timer counts five seconds; cutoff at seven seconds) or (b) too fast in the deliberate decision-making condition, using the same cutoff as in the intuitive decision-making condition (timer counts 30 seconds; cutoff at seven seconds). One hundred and ninety-eight participants (24.6%) were removed from the original sample. A floodlight and correspondence analysis supported the decision to use a cutoff of seven seconds to separate intuitive from deliberate decisions (see Supplementary materials).

### Procedure and measures

All online studies followed the same structure and used the same measures, except when otherwise stated. Participants were informed about the purpose and gave their consent in advance. First, participants saw an image of an autonomous sedan and learned about the capability of autonomous vehicles to drive without human intervention^1^. Next, participants were instructed that they would have control over the outcome of a moral dilemma that an autonomous vehicle faced and were familiarized with the visual elements of the dilemma (i.e., car, passenger, pedestrian, roadblock, and crosswalk) to increase their comprehension in the decision-making task. Finally, participants were instructed to assume the perspective and to respond within the given time according to their respective condition. In the moral decision-making task, participants were presented with the two illustrations representing the alternative outcomes of the dilemma and a timer counted the number of seconds left. Participants submitted their moral decision by clicking on the illustration that represented their preferred outcome. The decision-making task was followed by a manipulation check and a brief questionnaire on the participant’s beliefs, attitudes, and intentions towards autonomous vehicles. The demographic questions concerned the participants’ gender and age.

### Data analysis

We used SPSS 24 and R Studio (Version 1.1.423) for all analyses. Either χ^2^ or two-sided, independent samples t-tests were used to assess differences in group means. Binary logistic regression was used to regress the experimental conditions (perspectives, decision-making mode) and other control variables on participants’ decision to sacrifice the pedestrian.

## Results

**Figure 2:**
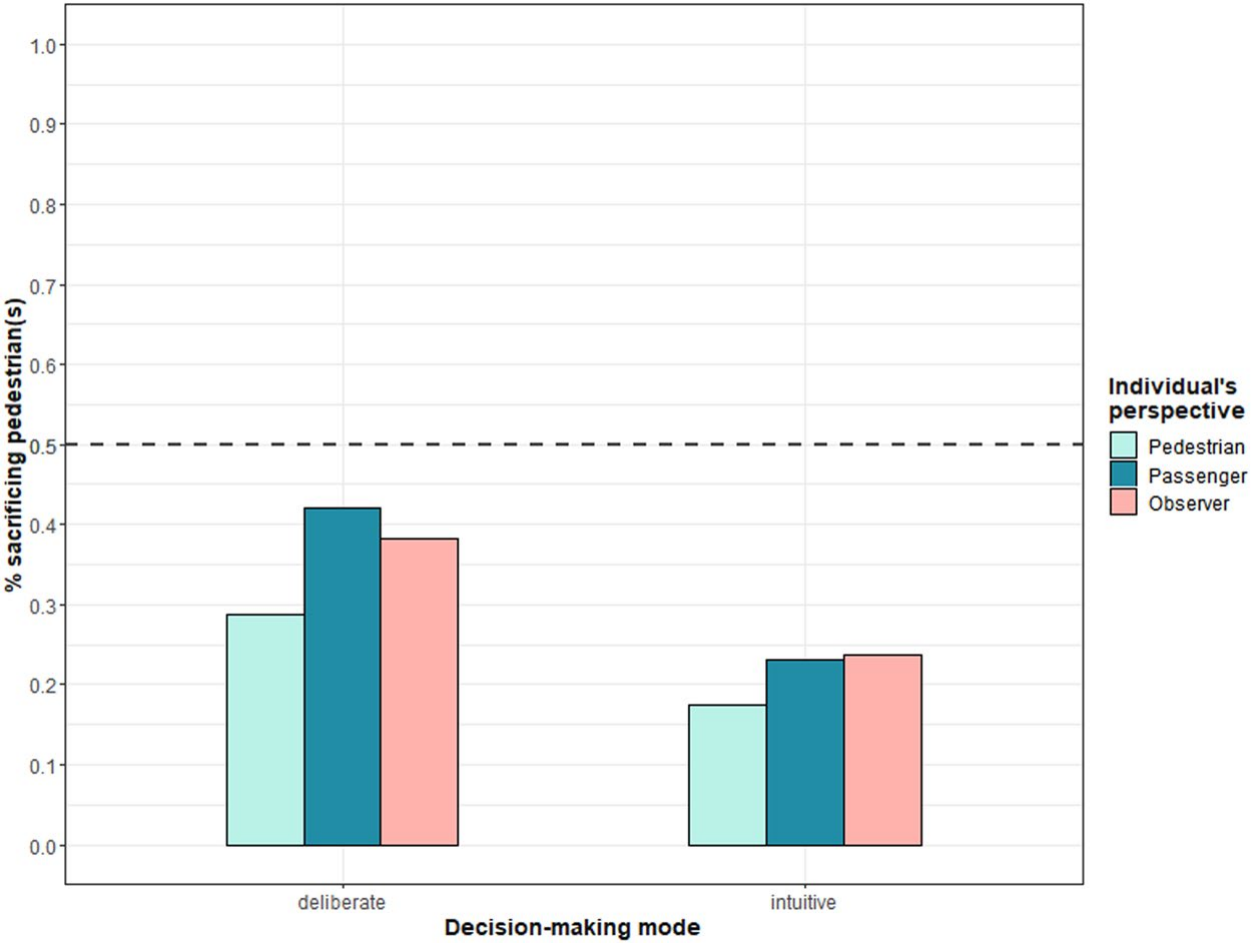
Moral decision to sacrifice the pedestrian by individual’s perspective and decision-making mode in Study 1. Caption: The dashed line marks the point at which lives of passenger(s) are valued equally to those of pedestrian(s).

First, we looked at the percentage of participants for each individual condition who decided that the autonomous vehicle in the one-versus-one dilemma should sacrifice the pedestrian. As shown in Fig. 2, participants’ decisions are anything but random and clearly trend towards sparing the pedestrian. Across the six conditions, participants’ moral decisions differ significantly (χ^27^ = 22.58, *p* < 0.003). The most notable difference can be observed between the two decision-making modes: the intuitive decision-making condition led to the pedestrian being sacrificed considerably less often (21.5%) than the deliberate decision-making condition (36.5%). The individual’s perspectives resulted in much smaller differences. Participants in the pedestrian perspective condition chose to sacrifice the pedestrian less often (22.8%) than participants in the passenger perspective condition (32.4%). In the control condition, the pedestrian was sacrificed in 30.7% of cases, on average.

Table 1 shows that intuitive decision-making results in a significant decrease in the likelihood of people sacrificing the pedestrian (*OR* 0.44, 95% CI: 0.30–0.65). The passenger perspective leads to a significant increase in sacrificed pedestrians relative to the pedestrian perspective (Perspective 1; *OR* 1.64, 95% CI: 1.01–2.65). Moral decisions in the control condition are not statistically different from the pedestrian perspective (see Perspective 2). We also tested for interactions of decision-making mode and personal perspectives and found no significant effects. In regard to control variables, females (*OR* 0.59, 95% CI: 0.40–0.89) and older participants (*OR* 0.78, 95% CI: 0.44–0.94) sacrificed the pedestrians significantly less often. Participants’ possession of a driver’s license and car use frequency did not significantly alter their moral decisions.

**Table 1:** Estimates for moral decision to sacrifice pedestrians in Studies 1–6.

Independent measure	Study 1			Study 2			Study 3			Study 4			Study 5			Study 6		
	*OR*	95% CI	*p*	*OR*	95% CI	*p*	*OR*	95% CI	*p*	*OR*	95% CI	*p*	*OR*	95% CI	*p*	*OR*	95% CI	*p*
***Main effects***																		
Decision-making mode (Intuitive vs. Deliberate)	0.44	0.30–0.65	<0.001	0.46	0.35–0.61	<0.001	0.37	0.27–0.52	<0.001	0.24	0.16–0.35	<0.001						
Perspective 1 (Passenger vs. Pedestrian)	1.64	1.01–2.65	0.045	1.81	1.27–2.57	0.001	1.86	1.25–2.79	0.002	1.80	1.14–2.86	0.012	1.64	0.81–3.32	0.168	1.55	0.95–2.53	0.077
Perspective 2 (Observer vs. Pedestrian)	1.55	0.95–2.53	0.077	1.48	1.04–2.10	0.029	1.40	0.93–2.10	0.103	1.57	0.98–2.50	0.059	1.00	0.49–2.06	0.999	1.14	0.69–1.87	0.619
***Situational factors***																		
Default path kills passenger				0.87	0.66–1.16	0.346												
Second passenger							1.93	1.37–2.73	<0.001									
Child as second passenger										1.06	0.94–1.20	0.333						
Removal of agency of passenger													2.23	1.24–4.03	0.007			
Norm violation 1 (Low vs. Control)																0.82	0.51–1.31	0.402
Norm violation 2 (High vs. Control)																0.61	0.37–0.99	0.047
***Control variables***																		
Females	0.59	0.40–0.89	0.011	0.57	0.43–0.77	<0.001	0.71	0.52–0.99	0.042	1.07	0.74–1.54	0.732	0.86	0.48–1.57	0.629	0.44	0.30–0.66	<0.001
Age (in years)	0.78	0.65–0.94	0.008	0.74	0.65–0.85	<0.001	0.71	0.61–0.83	<0.001	0.72	0.60–0.85	<0.001	0.69	0.53–0.91	0.009	0.99	0.97–1.00	0.115
Driver’s license	1.42	0.44–4.59	0.563	1.11	0.86–1.43	0.414	1.11	0.82–1.50	0.486	0.96	0.67–1.40	0.850	0.96	0.54–1.72	0.903	1.17	0.79–1.73	0.424
Car use frequency	1.04	0.89–1.22	0.610	1.04	0.94–1.14	0.488	1.00	0.89–1.13	0.956	0.92	0.80–1.06	0.225	1.06	0.80–1.40	0.694	1.03	0.88–1.21	0.711
Response time													1.06	1.03–1.08	<0.001	1.04	1.03–1.06	<0.001
***Model summary***																		
Constant	0.67		0.533	1.07		0.883	0.31		0.056	3.83		0.079	0.26		0.236	0.30		0.098
Observations	609			1,212			889			594			302			608		
*DF*	7			8			8			8			8			9		
Nagelkerke *R*^2^	0.10			0.10			0.13			0.17			0.19			0.14		

## Discussion

Study 1 demonstrates that people’s decisions in the simplified one-versus-one moral dilemma are influenced by their decision-making mode and personal perspective. First, people’s intuitive decisions appear to favor sacrificing the passenger. When people take more time to deliberate on the decision, the moral preference trends towards indifference between the life of the passenger and that of the pedestrian. The personal perspective also influenced people’s moral decisions. The difference is driven by the pedestrian and the passenger perspectives, which led to more selfish decisions, in that both spared their own lives more often compared to the control condition. Nevertheless, even the passenger perspective condition generally favored sacrificing the vehicle, which contradicts self-preservation as the underlying motivation. In the absence of a utility trade-off that would allow distinguishing a utilitarian from a deontological moral doctrine, we proceeded to test two alternative explanations on the action and status quo biases in people’s moral decisions before concluding this discussion. In Study 3, we address the hypothesis about the moral doctrine and its moderation of people’s decision-making mode.

## Study 2: Alternative Explanations

Study 2 tests two alternative explanations for people’s prevalent decision to sacrifice the passenger in the one-versus-one dilemma presented in Study 1. The first explanation is an action bias, in which people choose action (changing the path of the vehicle) over inaction (staying on the default path) even when the outcome of taking action is irrational^29,30^. The alternative explanation is a status quo bias, according to which decision-makers are more likely to preserve the *default* state of an outcome over change^11,31^. This would mean that people perceive sacrificing the passenger as the default state.

**Figure 3:**
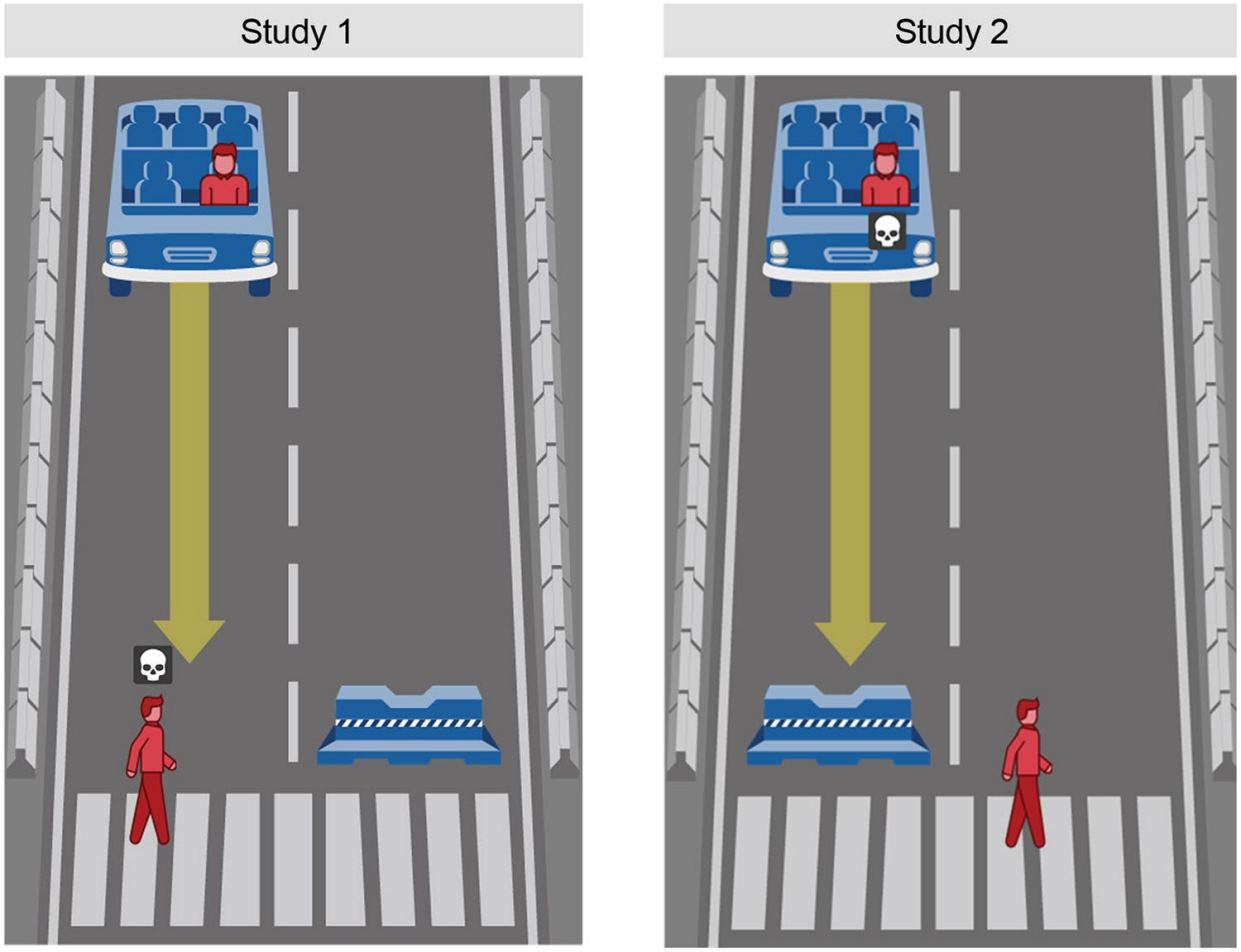
The default path of the vehicle in Studies 1 and 2. Note: Own work. Image assets adapted from the Moral Machine (http://moralmachine.mit.edu/) by Scalable Cooperation and MIT Media Lab [CC BY 4.0 (https://creativecommons.org/licenses/by/4.0/)].

We test these alternative explanations by inverting the outcome of the previously introduced one-versus-one dilemma (see Fig. 3). This time, instead of heading straight for the pedestrian, the vehicle is heading straight for the barrier, sacrificing the passenger. The alternative course of action in this adapted dilemma leads to the death of the pedestrian. An increase in sacrificed pedestrians would provide evidence for an action bias, as this would show consistency with people’s decisions to choose the alternative path in the first study. In contrast, a further increase in sacrificed passengers would support a status quo bias, assuming the decision to sacrifice the passenger represents the status quo. In the case of no change in the observed preferences, the findings would suggest that the observed trend to sacrifice a single passenger over a single pedestrian reflects an unbiased moral preference.

## Method

Participants (*N* = 848; 51.9% females; age: *M* = 33.02 years, *SD* = 11.81) were randomly assigned to the same 3 (perspective: pedestrian, passenger, observer) x 2 (decision-making mode: deliberate, intuitive) between-subject conditions introduced in Study 1. As shown in Fig. 3, the dilemma was adapted to feature the exact opposite outcomes of the dilemma used in Study 1. The remaining procedure was identical to Study 1, except that the questionnaire on participants’ beliefs about autonomous vehicles was shortened.

## Results and Discussion

We compared participants’ decision to sacrifice the pedestrian of Studies 1 and 2 (see Table 2) and found that in both alternative one-versus-one dilemmas, about one quarter of participants chose to sacrifice the pedestrian. Although the default paths in the two dilemmas lead to the exact opposite outcomes, the differences in people’s moral decisions in the individual conditions remain almost equal. The greatest deviation is seen in the control condition, which shifts from 38.1 percent to merely 29.1 percent in the deliberate decision-making condition. This suggests that people who were unbiased in their decision as to whom they would want to protect tended to spare the pedestrian even more. This shift appears only rational, since in Study 2, people would have to deliberately steer the vehicle to kill the innocent pedestrian.

**Table 2:** Percentage of sacrificed pedestrians in Studies 1 and 2.

Perspective	Study 1 (*N* = 609)		Study 2 (*N* = 603)	
	Intuitive	Deliberate	Intuitive	Deliberate
Pedestrian	17.5	28.7	17.0	24.2
Passenger	23.1	42.2	22.0	41.3
Observer	23.8	38.1	22.4	29.1

*Note*: The reported samples exclude participants who exceeded seven seconds in the intuitive decision-making condition and fell short of seven seconds in the deliberate decision-making condition.

In the logistic regression, reported in Table 2, we probe the difference in participants’ decisions that is attributed to the difference in the outcomes by pooling the independent samples of Studies 1 and 2 (*N = *1,212). This results in an increase in sample size and elevates the significance of the results of Study 1. The effect that we focus on, however, is the change in participants’ likelihood of choosing to sacrifice the pedestrian due to the default path being changed to driving into the barrier. As shown in Table 2, the effect of this alternative default path is not significant (*OR* 0.87, 95% CI: 0.66–1.16, *p* = 0.346). The result contradicts both alternative explanations (action bias, which expected the likelihood of sacrificed pedestrians to be increased, and status quo bias, which expected the opposite). We therefore conclude that a decision to sacrifice the passenger is unrelated to the *default* outcome of the dilemma and therefore likely represents a moral preference to avoid harming an innocent pedestrian in the street. Nevertheless, the simplified dilemma used in Studies 1 and 2 falls short in terms of creating a trade-off to determine the moral doctrine underlying people’s moral decisions. Study 3 follows up on this.

## Study 3: The Moral Doctrine

Study 3 builds on our previous findings and investigates people’s underlying moral decisions in a dilemma that is faced by an autonomous vehicle. To determine the presence of either a utilitarian or deontological moral doctrine, we adjust the previously introduced dilemma to become a closer representation of the original trolley dilemma^18^. That original dilemma, which has also been widely studied in the context of autonomous vehicle dilemmas^7,13^, presents people with a steep utility trade-off in choosing either to spare one person while sacrificing five others or kill one person to spare the lives of five. To introduce a utility trade-off in our dilemma, we therefore added a second passenger to the vehicle while keeping a single pedestrian in the crosswalk. Under the assumption that people employ a utilitarian moral doctrine, we would expect to see an increase of sacrificed pedestrians (in favor of saving two lives over one) compared with the previous one-versus-one dilemma. In the case of no change, the results would suggest that people employ a deontological moral doctrine motivated by the reasoning that it is simply not right to sacrifice an innocent person in the street.

## Method

The method in Study 3 was identical to that in our previous studies. Three hundred and ninety-three participants (50.6% females; age: *M* = 38.10 years, *SD* = 11.58) were recruited online and randomly assigned to the same 3 (perspective: pedestrian, passenger, observer) x 2 (decision-making mode: deliberate, intuitive) between-subjects conditions. The dilemma was based on that used in Study 2, but this time, it showed two passengers in the front seats of the autonomous vehicle and a single pedestrian in the crosswalk. The stimuli and instructions were adapted to reflect this change. The procedure remained identical to the previous studies.

## Results and Discussion

Table 3 shows participants’ moral decisions to sacrifice the pedestrian in Studies 2 and 3, which were identical except for the number of passengers in the autonomous vehicle. What is notable is that except for the intuitive decisions from the pedestrian perspective, the percentage of sacrificed pedestrians increases in Study 3 compared to Study 2. The largest difference is observed for the pedestrian perspective, in which the preference, for the first time in our studies, shifted in favor of sparing the passengers. In the intuitive condition, only 4.3 percent of participants chose to sacrifice the pedestrian, whereas in the deliberate decisions, this number increased to 60.0 percent. This large difference between the two decision-making modes illustrates the cognitive load that the moral decision task puts on participants, highlighting how time to deliberate can dramatically shift people’s moral preferences^16^.

**Table 3:** Percentage of sacrificed pedestrians in Studies 2 and 3.

Perspective	Study 2 (*N* = 603)		Study 3 (*N* = 286)	
	Intuitive	Deliberate	Intuitive	Deliberate
Pedestrian	17.0	24.2	4.3	60.0
Passenger	22.0	41.3	28.8	50.0
Observer	22.4	29.1	26.4	42.2

*Note*: The reported samples exclude participants who exceeded seven seconds in the intuitive decision-making condition and fell short of seven seconds in the deliberate decision-making condition.

In a logistic regression on the pooled, independent samples of Studies 2 and 3 (*N* = 889), reported in Table 1, we estimate the change in the likelihood of choosing to sacrifice the pedestrian that can be attributed the increase in the number of passengers. The results show that in the dilemma with two passengers, the likelihood of people sacrificing the pedestrian is 1.93 times higher than in the dilemma with the single passenger (*OR* 1.93, 95% CI: 1.37–2.73). This effect is highly significant (*p* < 0.001). The other experimental variables showed the same pattern seen in our previous studies.

The finding that the number of sacrificed pedestrians increases with the number of passengers is in line with previous research on the original trolley dilemma and in the context of autonomous vehicles^13^. The relatively higher utility of saving two lives over one appears to shift people’s moral decisions towards sacrificing the pedestrian relatively more often. This finding supports the hypothesis that people employ a utilitarian moral doctrine in a deliberate decision-making mode, while people in an intuitive, speedy decision-making mode are relying on the more accessible deontological moral doctrine. People’s moral preferences in the latter decision-making mode further reflect the internalized social norm that pedestrians in public roads may not be harmed by drivers, which US citizens are taught when they are young^32^.

While people’s moral decisions in the present study trended towards sparing the lives of the two passengers, the prevalent choice remained in favor of the single pedestrian. The moral doctrine that guides this decision appears to be stronger than the utility trade-off that was created in favor of the passengers in the two-versus-one dilemma. This raises the question of what degree of utility trade-off would be necessary for people to prefer that an autonomous vehicle actually harms an innocent pedestrian to avoid the certain death of its passengers. It is also of particular interest whether the difference between people’s moral decisions in intuitive versus deliberate decisions will increase or eventually converge.

## Study 4: The Value of Life

Study 4 builds on the previous finding that people’s moral decisions are influenced by the contextual factor of number of passengers in the presented dilemma. As seen in Study 3, the increased complexity of the dilemma (two-versus-one) leads to a larger difference in moral decisions between the two decision-making modes. In Study 4, we aim to further increase the complexity of the dilemma and thus the effects of decision-making mode and perspective by adding the passenger’s age as another contextual variable. In earlier research, age has been studied as a factor in moral dilemmas and connected to the value-of-life heuristic in people’s moral decision-making^13^. According to this research, the life of a younger person is valued over that of an older person, leading to a lower likelihood of sacrificing children in similar moral dilemmas. The recent publication of the global data of the Moral Machine experiment, however, limits this value-of-life observation to Western cultures; it shows that in Asian cultures, the trend is reversed (older people’s lives are more valued)^10^. Since we conducted this study in the US, we expect younger age to correspond to higher value and increase the decisions to spare the life of the younger person.

## Method

**Figure 4:**
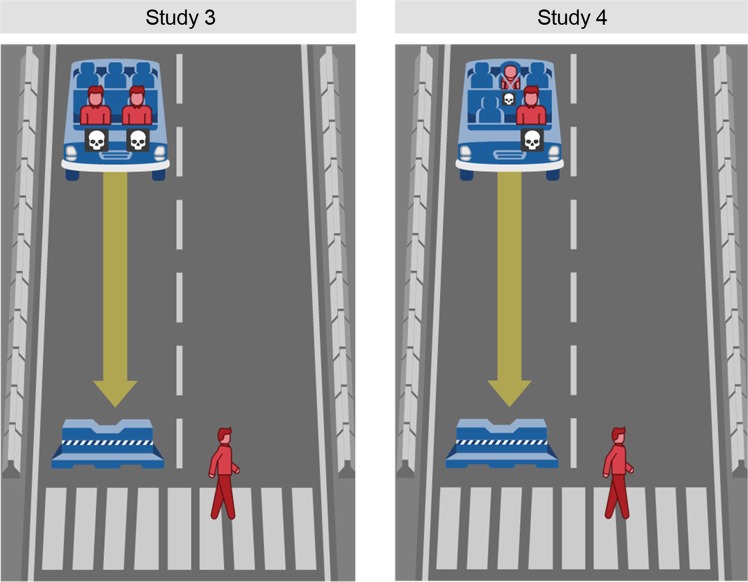
The default path of the vehicle in Studies 3 and 4. Note: Own work. Image assets adapted from the Moral Machine (http://moralmachine.mit.edu/) by Scalable Cooperation and MIT Media Lab [CC BY 4.0 (https://creativecommons.org/licenses/by/4.0/)].

Participants (*N* = 428; 50.5% females; age: *M* = 35.97 years, *SD* = 11.96), recruited on MTurk, were randomly assigned to the same 3 (perspective: pedestrian, passenger, observer) x 2 (decision-making mode: deliberate, intuitive) between-subject conditions as introduced in our previous studies. The dilemma was adapted from Study 3; in Study 4, two passengers were shown in the vehicle, one of whom was a child sitting in the back seat (see Fig. 4).

The stimuli and instructions were adapted to reflect the presence of the child in the car. The procedure remained identical to that in the previous studies. The questionnaire on participants’ demographics was extended to capture whether participants were parents of children similar in age to the child in the dilemma (0–9 years).

## Results and Discussion

First, we compare participants’ moral decisions observed in this study with the previous study that used an otherwise identical dilemma but showed an adult passenger instead of a child. The results, presented in Table 4, show that there is little difference in participants’ moral decisions to sacrifice the pedestrian. In fact, participants’ decisions in Study 4 are almost identical to those seen in Study 3, except for a change from 42.2 to 57.4 percent in sacrificed pedestrians in the deliberate decision-making condition in the observer perspective.

**Table 4:** Percentage of sacrificed pedestrians in Studies 3 and 4.

Perspective	Study 3 (*N* = 286)		Study 4 (*N* = 308)	
	Intuitive	Deliberate	Intuitive	Deliberate
Pedestrian	4.3	60.0	11.5	56.6
Passenger	28.8	50.0	34.4	50.0
Observer	26.4	42.2	34.0	57.4

*Note*: The reported samples exclude participants who exceeded seven seconds in the intuitive decision-making condition and fell short of seven seconds in the deliberate decision-making condition.

In a logistic regression on the pooled, independent samples of Studies 3 and 4 (*N* = 594), reported in Table 1, we determine the size of the effect that can be attributed to the difference of the child relative to the adult among the passengers in the two-versus-one dilemma. The model reveals that the child does not significantly increase the likelihood of people choosing to sacrifice the pedestrian (*OR* 1.06, 95% CI: 0.94–1.20, *p* = 0.333). This result suggests that people did not attribute a higher value to the child than to the adult passenger.

In a second logistic regression, shown in Table 5, we probe the influence of the control variable (children) and second-order interactions. Model 1 shows that intuitive decisions lead to a significant lower likelihood of participants sacrificing the pedestrian than deliberate decisions (*OR* = 0.30, CI: 0.18–0.49). The difference of the passengers’ perspective from the pedestrian is marginally significant (*OR* = 1.82, CI: 0.99–3.35), whereas the control is certainly not (*OR* = 1.69, CI: 0.90–3.18). When the interaction of perspective and decision-making mode is added (see Model 2, Table 5), the main effects of perspective diminish and the effect of decision-making mode becomes even stronger. Moreover, the results show that the likelihood of people’s intuitive decisions to sacrifice the pedestrian in the passenger perspective condition is four times higher than in the pedestrian perspective condition. The same trend is observed in the interaction of decision-making with the control relative to pedestrian perspectives; however, this effect is only marginally significant. This result suggests that people are less protective of the child in the pedestrian condition than in the passenger and control conditions.

**Table 5:** Estimates for moral decision to sacrifice pedestrians in Study 4.

Independent measure	Model 1			Model 2			Model 3		
	*OR*	95% CI	*p*	*OR*	95% CI	*p*	*OR*	95% CI	*P*
***Main effects***									
Decision-making mode (Intuitive vs. Deliberate)	0.30	0.18–0.49	<0.001	0.11	0.04–0.32	<0.001	0.09	0.03–0.30	<0.001
Perspective 1 (Passenger vs. Pedestrian)	1.82	0.99–3.35	0.053	0.98	0.43–2.24	0.955	0.74	0.28–1.94	0.539
Perspective 2 (Observer vs. Pedestrian)	1.69	0.90–3.18	0.104	1.04	0.44–2.45	0.922	1.18	0.42–3.27	0.754
***Interactions***									
Decision-making mode * Perspective 1				4.26	1.15–15.74	0.030	5.21	1.32–20.49	0.018
Decision-making mode * Perspective 2				3.29	0.85–12.77	0.085	3.44	0.81–14.55	0.093
Decision-making mode * Children							0.37	0.09–1.52	0.168
Perspective 1 * Children							1.97	0.38–10.32	0.422
Perspective 2 * Children							0.69	0.13–3.58	0.657
***Control variables***									
Children							0.70	0.19–2.50	0.582
Female							1.24	0.73–2.13	0.426
Age (in years)							0.72	0.56–0.92	0.008
Driver’s license							1.20	0.70–2.03	0.509
Car use frequency							0.89	0.73–1.08	0.231
***Model summary***									
Constant	0.83		0.446	1.21		0.538	7.66		0.032
Observations	308			308			308		
*DF*	3			5			13		
Nagelkerke *R*^2^	0.12			0.15			0.22		

*Note*: The analysis excludes participants who exceeded seven seconds in the intuitive decision-making condition and fell short of seven seconds in the deliberate decision-making condition.

Lastly, we test for the effect of children and interaction of children with the main effects. Model 3 shows no significant effect or interaction between parents of young children and the rest of the sample. This result suggests that parents do not project the consequences of the dilemma onto themselves and their own children. This, together with the mentioned findings, supports the theory that people’s consideration of the value of life in moral dilemmas is driven by deliberate decision-making, which in turn is associated with a utilitarian moral doctrine and absence of emotion^15^. Regardless, even in the deliberate decision-making mode, people’s decisions were only as good as flipping a coin for choosing whether one pedestrian versus two passengers should be sacrificed. The prevalent moral doctrine that deems it wrong to sacrifice an innocent pedestrian is surprisingly strong. Further increasing the utility trade-off would potentially shift the preference in favor of the passengers. In the following two studies, we therefore investigate the influence of agency (or illusion of agency) on people’s moral decisions in the autonomous vehicle dilemma.

## Study 5: Agency Bias

Study 5 tests another alternative explanation for people’s moral decisions, specifically whether the prevalent decision to sacrifice the passenger is caused by the inference of passenger agency. The concept of agency refers to humans’ capacity to change their immediate environment, shape the course of their lives, and control their actions^33^. In this and other research on the moral dilemmas of autonomous vehicles, the artificial intelligence drives the vehicle and is in control of the situation. Accordingly, agency can be attributed to the autonomous vehicle, however, not the passenger who is driven by one. Nevertheless, in visual representations of the dilemmas, the passenger often is in the driver’s seat, which, in a traditional, manually driven car, grants control over the vehicle and the outcome of the situation. Due to the human behavior of considering attribution of responsibility in moral decisions^34,35^, people’s attribution of agency could bias their moral decision. We test this hypothesis by comparing two otherwise identical dilemmas in which the passenger is in either the “driver’s” seat or the back seat. In both dilemmas, the passenger’s agency is the same, and we should not see a difference in people’s moral decisions. However, if the seating position facilitates a bias in the attribution of the passenger’s agency, we are likely to see a lower percentage of sacrificed passengers when the passenger is in the back seat.

## Method

**Figure 5:**
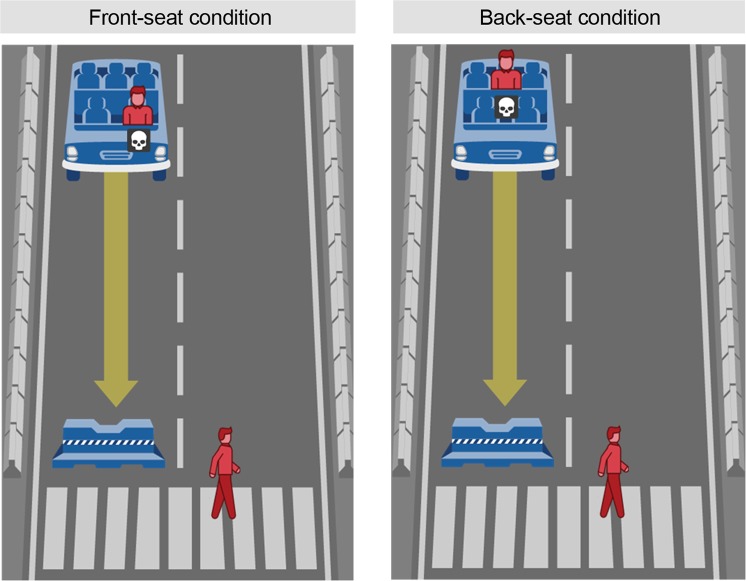
The default path of the vehicle for the front- and back-seat conditions in Study 5. Note: Own work. Image assets adapted from the Moral Machine (http://moralmachine.mit.edu/) by Scalable Cooperation and MIT Media Lab [CC BY 4.0 (https://creativecommons.org/licenses/by/4.0/)].

Participants (*N* = 302; 56.3% females; age: *M* = 37.05 years, *SD* = 12.06), recruited on MTurk, were randomly assigned to 3 (perspective: pedestrian, passenger, observer) x 2 (seating position: front, back) between-subjects conditions. We used the simple one-versus-one dilemma introduced in Study 2, but we varied the passenger’s seating position. In the front-seat position condition, the passenger was in the left front seat. Note that this is the same seating position that was used in all our previous studies and that the illustration does not show a steering wheel. In the back-seat position condition, the passenger was in the middle back seat (see Fig. 5). The three perspective conditions remained identical to our previous studies. Participants were instructed that the vehicle was driven autonomously and that they were asked to choose the more appropriate outcome.

Because the decision-making mode was not actively manipulated in this study, we controlled for response time in the logistic regression model. No participants were excluded from the analysis.

## Results and Discussion

Table 6 shows the percentage of participants that chose to sacrifice the pedestrian for each of the six conditions. In all back-seat conditions, participants sacrificed the pedestrian more often (sparing the life of the passenger). The difference is lowest in the observer (control) condition and more than doubles in the passenger perspective. Pedestrian and passenger perspectives are nearly the same in the front-seat condition and differ largely in the back-seat condition.

**Table 6:** Percentage of sacrificed pedestrians in Study 5 (*N* = 302).

Perspective	Seating position	
	Front	Back
Pedestrian	15.7	30.0
Passenger	18.4	44.9
Observer	25.5	28.8

The logistic regression on the Study 5 sample, reported in Table 2, finds that the passenger’s seating position in the back seat results in a 2.23 times higher likelihood of the pedestrian being sacrificed relative to the front-seat position (*OR* 2.23, 95% CI: 1.24–4.03). The effect is highly significant *(p* = 0.007) and supports the hypothesis that people’s moral decisions are affected by the inference of the passenger’s agency. This result suggests that people, despite our stressing of the implications of an autonomous vehicle, are biased by attribution of agency, because they sacrifice the passenger more often when the passenger is in the “driver’s” seat. A potential mechanism for this agency bias is found in previous research on the attribution of responsibility based on the proximity to the immediate cause of action^36,37^ that would explain why the passenger in the driver’s seat is perceived more responsible for the actions than the passenger in the backseat.

## Study 6: Social Norms Violation

Study 6 investigates the influence that social conventions, and specifically their violation, have on people’s moral decisions^38^. Social norms are conceptualized as rules that are shared by members of society, guide social behavior, and serve to coordinate societies^39^. In particular, we are interested in the effect that violating the social norm of not endangering others in traffic situations has on people’s decisions to sacrifice the pedestrian in the previously used one-versus-one moral dilemma. We added two alternative versions of the same dilemma, in which the pedestrian violates traffic norms by walking out in front of a car and jaywalking at a red light. We compare the results of the two norm conditions with the default dilemma, in which the pedestrian walks in the crosswalk, as expected by the social norm. We expect norm violations to increase the likelihood of the pedestrian being sacrificed, irrespective of people’s perspective.

## Method

**Figure 6:**
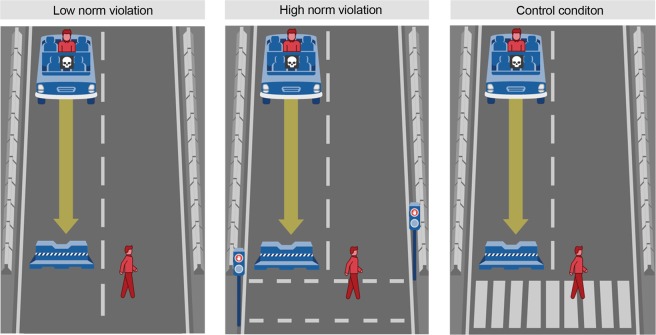
The default path of the vehicle for the norm violation conditions in Study 6. Note: Own work. Image assets adapted from the Moral Machine (http://moralmachine.mit.edu/) by Scalable Cooperation and MIT Media Lab [CC BY 4.0 (https://creativecommons.org/licenses/by/4.0/)].

Participants (*N* = 608; 59.4% females; age: *M* = 35.07 years, *SD* = 11.20) were randomly assigned to 3 (perspective: pedestrian, passenger, observer) x 3 (norm violation: low, high, control) between-subject conditions. The perspective conditions were manipulated in the same way as in the previous studies. The norm violation conditions consisted of low norm violation, high norm violation, and no norm violation (control) (see Fig. 6). In the low norm violation condition, the pedestrian walked in a street where there were no signs, traffic lights, or crosswalk. In the high norm violation condition, the pedestrian jaywalked at a red light. In the control condition, the pedestrian walked in the crosswalk, as in all previous studies. Again, all participants were included in the analyses, and the logistic regression model accounts for the effect of decision-making modes by controlling for participants’ response times.

## Results and Discussion

Table 7 shows the percentage of participants that chose to sacrifice the pedestrian for each of the nine conditions. The results show that participants sacrificed the pedestrian less in the high norm violation condition than in the low norm violation and control conditions. The moral decisions in the control condition replicate the pattern observed in earlier studies.

**Table 7:** Percentage of sacrificed pedestrians in Study 6 (*N* = 608).

Perspective	Norm violation		
	High	Low	Control
Pedestrian	18.8	25.8	29.9
Passenger	32.4	24.2	38.6
Observer	23.8	29.6	26.5

The logistic regression on the Study 6 sample, reported in Table 2, contrasts both norm violation conditions with the control condition. The result shows that high norm violation (jaywalking) significantly reduces the likelihood of sacrificing the pedestrian (*OR* 0.61, 95% CI: 0.37–0.99, *p* = 0.047). The low norm violation, on the other hand, shows no significant effect on participants’ moral decisions (*OR* 0.82, 95% CI: 0.51–1.31, *p* = 0.402). This finding contradicts our hypothesis that norm violation would increase the likelihood of sacrificing the pedestrian. This result required further investigation; when we examined the stimuli, it became apparent that the traffic light in the high norm violation condition might have been interpreted as governing the autonomous vehicle instead of the pedestrian. In that case, the norm violation would be committed by the autonomous vehicle, which could explain the unexpected result. Due to this controversy, we revisit the hypothesis with an improved, unambiguous stimulus for manipulation of the norm violation in Study 7.

## Study 7: Systematic Replication

Study 7 revisits our previous hypotheses and replicates the effect of decision-making mode and personal perspective in a controlled lab experiment. Its objective is to increase the validity of our previous findings by controlling for the experimental conditions and replicating the findings in a different sample population. Besides the main manipulation of participants’ perspectives and decision-making mode, Study 7 tests four different situational factors, which were individually examined in Studies 2, 3, 4, and 6: the vehicle’s alternative default path (Study 2), the number of passengers (Study 3) and pedestrians, the presence of a child among the passengers (Study 4) and pedestrians, and a social norm violation by a pedestrian (Study 6). The agency bias addressed in Study 5 was not actively manipulated because the passengers’ seating positions depended on the number of passengers in the vehicle. In line with our previous studies, we expected that the alternative default path would not change participants’ moral decisions and that the number of passengers would increase the likelihood of sacrificing pedestrians and vice versa. Likewise, the presence of a child among the passengers would increase the likelihood of sacrificing pedestrians and vice versa. The pedestrian’s norm violation was expected to result in an increase of sacrificed pedestrians, in contrast to our finding in Study 6.

## Method

### Participants

One hundred and twenty-eight participants aged between 19 and 62 years (*M* = 25.46, *SD* = 6.42; 64.8% females) were recruited through the subject pool at a behavioral science lab in Denmark. Participants received 100 DKK (~15.78 USD) on completion of the study. The experiment ran continuously, and data collection was completed in one week.

### Stimuli

We created 56 visual representations of the two alternative outcomes (sacrifice pedestrian[s], sacrifice passenger[s]) of a fractional factorial design of 28 dilemmas combining variations of the five experimental situational factors (alternative default path, number of passengers [1, 2, or 4], number of pedestrians [1, 2, or 4], child among passengers, child among pedestrians, and social norm violation of pedestrian; see Supplementary materials). The fractional factorial design was computed in JMP. The stimulus for illustrating the pedestrian’s norm violation (jaywalking) was adapted from Study 6 and proved to allow an unambiguous interpretation of the red traffic lights (see Supplementary materials). The adjusted illustration showed three traffic lights, two red pedestrian traffic lights and a green traffic light for the vehicle, with the pedestrian walking in the middle of the street.

### Design

The experimental design for this study was a 2 × 3 × 28 mixed design, with decision-making mode (intuitive, deliberate) as the between-subjects factor and perspectives (pedestrian, passenger, control) and dilemmas (factorial design of five situational factors) as the within-subjects factors. Participants were randomly assigned to the decision-making mode and always started with the control perspective before proceeding to the two personal perspectives in randomized order. The presentation of the dilemmas was randomized for each perspective. Three perspectives with 28 dilemmas each totaled 84 moral decisions for each participant.

The decision-making mode was manipulated by means of time pressure (<5 seconds response time for intuitive decisions, <60 seconds response time for deliberate decisions) and aided by a countdown timer. The manipulation of time pressure resulted in significant differences in participants’ reported level of feeling pressured in the intuitive (*M* = 5.09, *SD* = 1.38) versus deliberate (*M* = 4.04, *SD* = 1.90) decision-making mode conditions (t(126) = −3.54, *p* < 0.001, 95% CI: −1.629, −0.462). Participants adapted to the effect of time pressure in that the reported level of feeling pressured is lower after the personal perspective conditions (passengers: *M* = 4.81, *SD* = 1.77; pedestrians: *M* = 4.90, *SD* = 1.60) compared to the control condition (*M* = 5.55, *SD* = 1.25). In contrast to the previous online studies, participants were only allowed to enter their choice within the time limit. When the timer ran out, the dilemma was skipped and participants were reminded to answer within the given time limit; skipped dilemmas repeated after a full set of 28 dilemmas, in randomized order, until decisions were successfully recorded for all dilemmas. Presentation order did not influence people’s moral decisions (*OR* = 1.00, 95% CI: 0.89–1.11, *p* = 0.963).

### Measures

Participants’ moral decisions were recorded as their choice to sacrifice the pedestrian(s) or passenger(s) for each presented dilemma. Demographic and control variables included age, gender, education, residential area (city center, suburbs, countryside), use frequency of transportation means (bike, bus, car, train, walking, and airplane; measured on 5-point scales, 1 = “never,” 2 = “few times a year,” 3 = “few times a month,” 4 = “few times a week,” 5 = “daily”), possession of a driver’s license (yes, no), car ownership (none, one, two or more), experience with autonomous vehicles (yes, no) and knowledge of the term “autonomous vehicle” (yes, no). The postquestionnaire also included a series of exploratory measures unrelated to the purpose of this research (see Supplementary materials).

### Procedure

Approximately one week before the lab study, two hundred and eighteen participants (64.22% females; age: *M* = 25.39, *SD* = 6.27) filled out a short prequestionnaire. Participants evaluated the morality of six traditional, text-based dilemmas: three moral-personal and three moral-impersonal dilemmas (see Supplementary materials). The moral-impersonal dilemmas included the original trolley dilemma, in which a rail worker must decide whether to pull a lever that would kill one innocent person to save five trolley passengers^21^; the moral-personal dilemmas included the original (foot-)bridge dilemma, in which the decision-maker must choose whether to push an innocent fat man in front of a trolley to save five people^18^. Participants’ moral judgements in these dilemmas served as a measure of their general morality in personal and non-personal dilemmas, which was entered as a control variable in the logistic regression analysis of the main study. All participants who successfully completed the pre-study were invited to the lab.

In the main experiment, participants of the same decision-making mode were grouped in batches to minimize noise and distraction due to a large difference in experiment duration (up to 15 minutes). The experiment started only after all participants were seated and ready. First, participants learned about the features of autonomous vehicles and were familiarized with the elements (i.e., car, pedestrian, passenger) used for the visual representation of the dilemmas (see Supplementary materials). To become familiar with the mechanics of responding under time pressure, participants in the intuitive decision-making condition then completed six non-dilemma-related training trials, in which the task was to select the illustration that showed more circles. Participants were then instructed to assume the observer perspective and respond to the first set of 28 dilemmas. Participants’ decisions were recorded as a selection of the outcome that they found more appropriate. An artificial loading screen of seven seconds in between dilemmas was used to reduce interference with moral decisions due to cognitive load^19^. In between perspectives, participants were forced to pause for another 60 seconds. After completing all three perspectives, participants completed a postquestionnaire containing measures on their beliefs, attitudes, and intentions towards autonomous vehicles.

### Data analysis

All analyses were conducted only after completion of the data collection. Two-sided, independent samples t-tests were used to assess differences of group means. Binary logistic regression was used to regress the experimental conditions (decision-making mode, perspective, situational factors) onto participants’ decision to sacrifice the pedestrian. All participants were included in the analyses.

## Results

**Figure 7:**
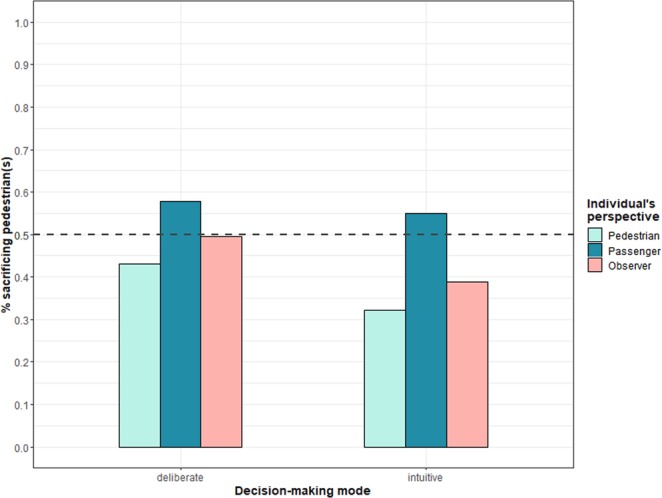
Moral decision to sacrifice the pedestrian by individual’s perspective and decision-making mode in Study 7. Note: The dashed line marks the point at which lives of passenger(s) are valued equally to those of pedestrian(s).

Figure 7 shows the distribution of moral decisions for the six decision-making modes by perspective conditions collapsed over the 28 dilemmas. In the control condition, pedestrians were sacrificed as often as passengers in the deliberate decision-making mode condition (*M* = 49.62%, *SD* = 50.01%) and less often in the intuitive decision-making mode condition (*M* = 38.75%, *SD* = 48.73%). In the pedestrian perspective condition, the pedestrian was sacrificed less on average, with a difference between the deliberate (*M* = 43.13%, *SD* = 49.54%) and intuitive (*M* = 32.14%, *SD* = 46.72%) decision-making mode conditions. The passenger perspective was the opposite, with more pedestrians sacrificed in both the deliberate (*M* = 57.85%, *SD* = 49.39%) and intuitive (*M* = 54.90%, *SD* = 49.77%) decision-making mode conditions. The difference between the two decision-making mode conditions is smaller in the passenger perspective condition than in the pedestrian perspective and control conditions.

Table 8 shows the logistic regression model of Study 7. In line with Study 1, the result shows that intuitive decision-making significantly lowers the likelihood deciding to sacrifice the pedestrian (*OR* 0.71, 95% CI: 0.65–0.79). The likelihood of sacrificing the pedestrian is significantly higher in the passenger perspective (Perspective 1; *OR* 2.87, 95% CI: 2.57–3.21) and control (Perspective 2; *OR* 1.47, 95% CI: 1.20–1.79) conditions relative to the pedestrian perspective condition. Second, all situational factors of the dilemmas result in significant and relatively large effects on people’s moral decisions. In line with Study 2, the alternative path of the vehicle does not alter the decision of the two outcomes (*OR* 1.08, 95% CI: 0.99–1.18, *p* = 0.095). In line with Study 3, a larger number of passengers increases the likelihood of sacrificed pedestrians – likewise, a larger number of pedestrians increases the likelihood of sacrificed passengers. In line with Study 4, the presence of children among the passengers significantly increases the likelihood of sacrificed pedestrians – likewise, the presence of children among the pedestrians increases the likelihood of sacrificed passengers. And finally, in line with the initial hypothesis of Study 6, the pedestrian’s norm violation results in a large and significant increase in the likelihood of being sacrificed (*OR* 3.63, 95% CI: 3.31–3.98). This finding supports our explanation that the result obtained in Study 6 was likely due to misinterpretation of the stimuli.

**Table 8:** Estimates for moral decision to sacrifice pedestrians in Study 7.

Independent measure	*OR*	95% CI	*p*
***Main effects***			
Decision-making mode (Intuitive vs. Deliberate)	0.71	0.65–0.79	<0.001
Perspective 1 (Passenger vs. Pedestrian)	2.87	2.57–3.21	<0.001
Perspective 2 (Observer vs. Pedestrian)	1.47	1.20–1.79	<0.001
***Situational factors***			
Alternative default path	1.08	0.99–1.18	0.095
Number of passengers 1 (Two vs. One)	2.24	1.95–2.56	<0.001
Number of passengers 2 (Four vs. One)	4.59	4.08–5.16	<0.001
Number of pedestrians 1 (Two vs. One)	0.52	0.46–0.59	<0.001
Number of pedestrians 2 (Four vs. One)	0.21	0.19–0.23	<0.001
Child among passengers	1.50	1.37–1.65	<0.001
Child among pedestrians	0.66	0.60–0.72	<0.001
Norm violation of pedestrian	3.63	3.31–3.98	<0.001
***Control variables***			
Female	0.95	0.86–1.06	0.374
Age	0.98	0.97–0.99	<0.001
Driver’s license	1.64	1.43–1.87	<0.001
Car use frequency	0.89	0.83–0.94	<0.001
Car ownership	0.81	0.76–0.87	<0.001
Education 1 (College vs. High School)	1.22	1.05–1.41	0.009
Education 2 (University vs. High School)	1.13	1.00–1.29	0.059
Region 1 (Suburb vs. City Center)	0.98	0.89–1.08	0.700
Region 2 (Countryside vs. City Center)	3.29	2.37–4.55	<0.001
Knowledge	1.16	1.01–1.34	0.042
Experience	0.55	0.46–0.67	<0.001
Presentation order	1.00	0.89–1.11	0.963
Text-based dilemma Personal	1.14	1.06–1.23	<0.001
Text-based dilemma Non-Personal	1.00	0.95–1.05	0.942
***Model summary***			
Constant	0.41		<0.001
Observations	10,752		
*DF*	25		
Nagelkerke *R*^2^	0.34		

In regard to control variables, results show that participants’ age, car usage frequency, car ownership and experience with autonomous vehicles significantly decrease the likelihood of sacrificing the pedestrian. Possession of a driver’s license, knowledge of autonomous vehicles, higher education, and living in the country, on the other hand, significantly increase the likelihood of participants sacrificing the pedestrian. Participants’ judgement of the appropriateness of sacrificing a single person to save many in the moral-personal text-based dilemmas shows a significant increase in the likelihood of sacrificing the pedestrian (*OR* 1.14, 95% CI: 1.06–1.23).

## Discussion

The findings of our study address all the previous hypotheses and provide converging validity on the effects found in the previous studies. First and foremost, the findings demonstrate a strong and significant effect of decision-making mode on people’s moral decisions, thus supporting the evidence of previous research^16^. The effect shows that regardless of perspective and situational factors, people express an intuitive moral preference to sacrifice the autonomous vehicle and its passengers rather than harming pedestrians. Second, the results provide evidence that personal perspective significantly changes the moral decision on who should be sacrificed^7^. As discussed by Bonnefon, *et al*.^7^, the differences in perspective contribute to a social dilemma in the moral programming of machines, as passengers favor sacrificing pedestrians and vice versa. Our study supports the notion of a personally biased morality and further shows that the perspective of the observer lies in between the two personally motivated perspectives.

In addition to these two experimental factors, Study 7 offers a plethora of information on the influence of situational factors. In line with our findings from Study 2, people appear to show neither status quo nor action bias. In line with the hypothesized influence of utility on people’s moral decisions, the findings clearly show the utilitarian doctrine being applied. That is, people maximize the utility of lives saved by sacrificing the group with fewer people. Moreover, people consider age in the utility function and tend to spare the lives of children over those of adults. This further confirms the influence of a value-of-life heuristic in people’s moral decision-making, as shown by Sütfeld, *et al*.^13^. Lastly, in line with our original hypothesis in Study 6, people’s decisions seem to be strongly affected by the pedestrian’s norm violation. As shown in this study, regardless of all other factors, pedestrians who violate the norm of stopping at a red light are sacrificed considerably more. This influence further caters to the cultural embeddedness in people’s moral decisions: the violation of a norm, such as obeying traffic regulations, might be punished less severely in certain societies. Likewise, the valuation of utility and the value of life attached to it appear to be bound to cultural views, as highlighted by the large cross-country sample of the Moral Machine experiment^10^.

## Robustness of Results

**Figure 8:**
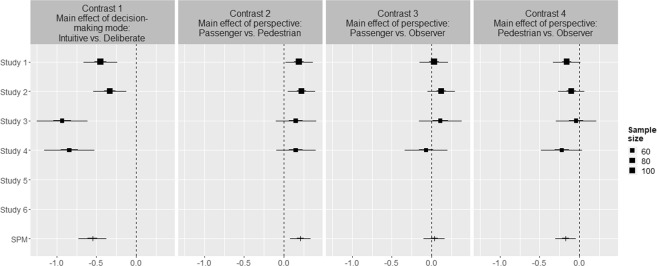
Main effects estimates on moral decision to sacrifice the pedestrian(s) for Studies 1–6. Note: Effect estimates are given by the squares for single-study estimates and the horizontal bars for SPM estimates; 50% and 95% intervals are given by the thick and thin lines, respectively. The average sample size per condition in each study is given by the size of the squares. The vertical dotted lines indicate the null-effect.

**Figure 9:**
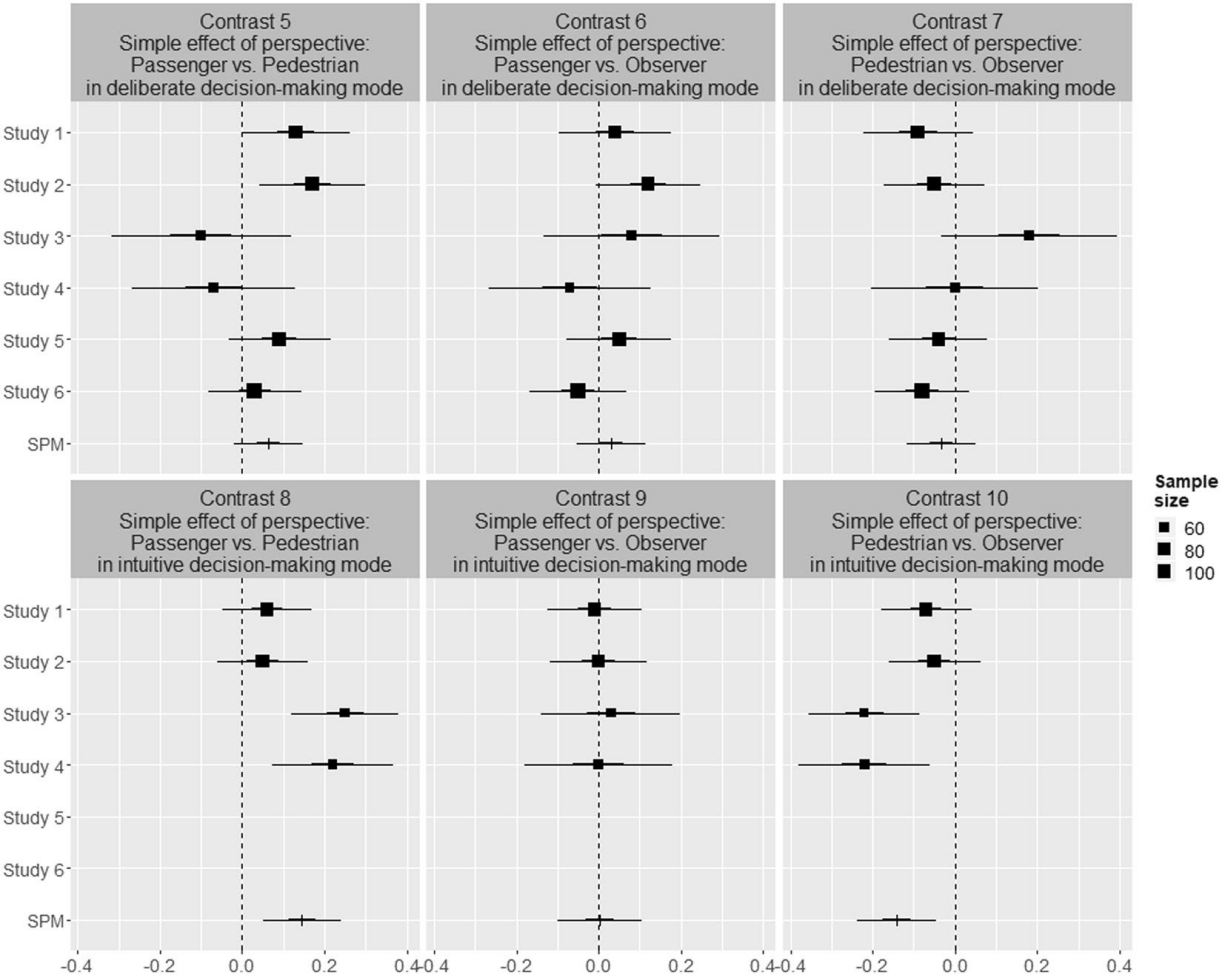
Simple effects estimates for moral decision to sacrifice the pedestrian for Studies 1–6. Note: Effect estimates are given by the squares for single-study estimates and the horizontal bars for SPM estimates; 50% and 95% intervals are given by the thick and thin lines, respectively. The average sample size per condition in each study is given by the size of the squares. The vertical dotted lines indicate the null-effect.

We subjected our data to an internal meta-analysis^40^ to validate our findings on the effect of decision-making mode and personal perspective on people’s moral decisions for Studies 1–6. Study 7 was not included in the analysis because it featured a substantially different design, which, in contrast to the almost identical online studies, introduced too much variation across its 168 different conditions. Figure 8 shows the results of the main effects of decision-making mode and perspective. The simple effects are shown in Fig. 9. Summary information and coding of the contrast are provided in the Supplementary materials. The *I*^2^ was estimated at 69.90% (95% CI: 54.75%, 79.98%), suggesting that heterogeneity across the studies is high. This was expected due to the considerable variation of situational factors across the online studies.

As shown in Fig. 8, the experimental factor of intuitive decision-making significantly reduces the likelihood of sacrificed pedestrians, with an overall effect size of −0.54 (95% CI: −0.72 to −0.38; see Contrast 1). This effect is consistent with the finding in Study 7. Moreover, the results show a significant increase in sacrificed pedestrians for people in the passenger relative to the pedestrian perspectives, with an overall effect size of 0.21 (95% CI: 0.08–0.34; see Contrast 2). Again, this effect is consistent with the findings in Study 7. Similarly, in line with Study 7, the observer (control) perspective lies in between the pedestrian and passenger perspectives. The observer perspective is not statistically different from the passenger perspective (0.03 [95% CI: −0.10–0.17]; see Contrast 3) but is statistically different from the pedestrian perspective (−0.17 [95% CI: −0.30 to −0.05]; see Contrast 4). This finding aligns with results obtained in Study 7 and suggests that the more accessible perspective, even when people are asked only to observe the situation, is that of the passenger.

Figure 9 shows that the simple effects of personal perspective in the intuitive decision-making (Contrasts 8–10) and deliberate decision-making (Contrasts 5–7) mode conditions replicate the direction of the main effect of perspective (Contrast 2–4). However, considerable variation can be observed across the six studies, which can be attributed to the studies that featured dilemmas with increased complexity (Studies 3 and 4). Moreover, the significant difference between perspectives in the online studies appears to be driven by the intuitive decision-making mode, as the results show that the perspectives are not significantly different in the deliberate decision-making mode conditions.

Taken together, these estimates, along with the visual convergence of effects, obtained in the internal meta-analysis are particularly reassuring because they reflect the consistency of the findings of the online studies (Studies 1–6) with those of the lab study (Study 7). More information, including contrast estimates and 95-percent intervals, is enclosed in the Supplementary materials.

## General Discussion

The present research updates the current knowledge on people’s morality in dilemmas faced by autonomous vehicles. The reported studies demonstrate that people’s moral decisions, regardless of the presented dilemma, are biased by their decision-making mode and personal perspective. Under intuitive moral decisions, participants shift more towards a deontological doctrine by sacrificing the passenger instead of the pedestrian. Once the personal perspective made salient participants preserve the lives of that perspective, i.e. the passenger shifts towards sacrificing the pedestrian, and vice versa. These effects are supported by a combined pool of more than ten thousand individual moral decisions across seven studies, which consistently find that the two moral decision-making biases cause substantial variations in people’s decisions.

The most prevalent effect – the distinct decision-making mode – is moderated by cognitive processing time. The less time people were permitted, the more their decisions trended towards a deontological moral doctrine. With the ability to process a given dilemma deliberately, people’s decisions trended towards a more utilitarian doctrine. Further, the investigation of people’s individual perspectives highlights that deliberately priming people to think as observers when judging moral dilemmas does not necessarily remove bias from their decisions. The results show that people’s decisions in the control condition (instructed to use the perspective of an observer) are almost identical with those in the passenger perspective. While this finding can be explained by the majority of our sample being frequent car users, it highlights that moral decisions gathered from representative samples of the US population are likely going to overrepresent the passenger perspective and lack the pedestrian perspective. As a result, personal perspectives represent a major bias in people’s moral decisions and underline the social challenge in the design of an universal moral code for autonomous vehicles^7^.

Our research further validates as well as extends previous findings on people’s moral decisions that have been identified as global moral preferences and those guided by a series of readily accessible and culturally acquired mental shortcuts^10^. First, our results show that people tend to maximize the number of saved lives, even if an innocent pedestrian is sacrificed in the process. This consistent pattern in the preference to save the lives of the many supports the global moral preference observed in the Moral Machine experiment^10^ and reflects the utilitarian moral doctrine in terms of the original trolley problem^18^. Second, our research provides further evidence that the life of a younger person is valued over that of an older one – as was shown to be the case in Western cultures, including the US and Denmark^10^. This moral decision appears to be independent of the decision-maker being a parent. In addition, our findings show that the prevalent moral doctrine that deems it wrong to sacrifice an innocent pedestrian is surprisingly strong. In fact, results show the limitation of this consideration in that the younger life of a passenger is not necessarily valued over a single innocent adult pedestrian. Lastly, the results of our studies show that when a social norm is violated (i.e., pedestrians jaywalk in front of the autonomous vehicle), people trend towards favoring a deontological doctrine and collectively punish the norm violation. This reflects the moral preference to spare the lawful, also found in the Moral Machine experiment^10^. A complication in the inference of collectively perceived norm violations highlighted by our results is that people are biased towards overestimating the attribution of agency of the passengers.

### Implications

From a theoretical perspective, this research provides supporting evidence for the presence of dual-process theory in people’s moral decision-making. It demonstrates not only that two states of decision-making modes can be achieved by limiting the available time to process decisions but also that the response time itself – in the absence of experimental manipulation – results in a significant difference in people’s moral decisions. In line with in earlier studies, the distinct route towards making the moral decision is found to alter peoples’ use of moral doctrines (deontological vs. utilitarian) which are associated with deliberate thinking (for utilitarian moral decisions) and intuitive thinking (for deontological moral decisions)^13,16,27^. In addition, this research contributes to the theory of bounded rationality by demonstrating that people’s consideration of a personal perspectives in moral dilemmas of autonomous vehicles leads to biased moral decisions that favor positive outcomes for their perspective.

From a practical standpoint, this research addresses critical aspects in the approach of inferring moral guides for autonomous vehicles from people’s moral decisions. For manufacturers of autonomous vehicles and ambitious projects such as the Moral Machine experiment, these findings on human decision-making biases imply that sourcing people’s moral preferences on a large scale requires developing a standardized and reliable instrument that actively controls for participants’ personal perspective and decision-making mode. The measurement of universal moral guidance should evenly balance moral decisions from all stakeholders who are directly affected by the outcome of the vehicle’s decision. Moreover, the instrument should force participants into one of the two decision modes, as this step primarily determines the moral doctrine that will be used. This decision, of course, can be moral but also strategic. If a manufacturer aims to emphasize more norm-driven, emotional responses, this can be easily achieved by limiting people’s cognitive processing time.

For policy makers, this research offers an interesting insight into the moral trade-off between people’s rationale of utility and social norms. It suggests that the expectation of moral-acting autonomous vehicles implies that while they should make computed decisions to maximize the good of society, they should simultaneously take situational factors into account. In this context, people seem to expect autonomous vehicles to become an agent that enforces the widely accepted social norms (i.e., obeying traffic regulations) and therefore favors punishing norm violations in critical incidents. This further implies that the development of moral autonomous vehicles must be closely aligned with the accepted (or enforced) norms.

### Future research

While these findings may be very useful to researchers and companies interested in understanding the moral decision-making biases of people in the realm of autonomous vehicle dilemmas, the present research has limitations that warrant discussion and offer avenues for future research.

First, the dilemma and the variations of it used in this and other research in this field represent modern versions of the original trolley problem and so largely exaggerate the moral decisions that autonomous vehicles will face in their everyday routines. While previous fatal incidents with autonomous vehicles highlight the importance of this extreme scenario^41^, we suggest that future research should focus on the more practical and more likely dilemmas that occur in everyday use, including the degree to which an autonomous vehicle should be able to be more aggressive when maneuvering in high traffic conditions or overtaking slow vehicles on the highway, speed to a destination if the passenger has a serious condition or needs to catch a connection, and adapt to the preferences or commands of its user, such as disregarding traffic regulations or roaming the streets without a specific destination.

Second, the dilemmas studied use definitive outcomes for the two alternatives – the passengers or pedestrians will die, while the others live. While this is the default assumption of the trolley dilemma and contrasts the possible extremes, in the real world, the odds would never be perfectly even. In fact, it is reasonable to assume that future automated, high-tech vehicles will provide superior safety mechanisms to protect passengers against potential incidents or software malfunctions and that passengers therefore are more likely to survive a critical incident. To create a better and more realistic picture, it is important that future studies provide decision-makers with a more realistic distribution of the probabilities of survival in moral dilemmas^11^.

Lastly, this research investigates the moral decision-making bias that is attributed to people’s personal perspectives. While we find a consistent pattern that is independent of various situational factors, future research could benefit greatly from an investigation of the motivations behind individuals’ moral decisions in the context of the use of autonomous vehicles. It would be particularly interesting to study to what degree people who are biased by a certain personal perspective are willing to accept a different, possibly opposing, perspective. Testing interventions that move people to voluntarily agree to a common, universal moral doctrine would be of interest to practitioners and researchers in this field.

### Ethical statement

All methods were carried out in accordance with relevant guidelines and regulations. Written informed consent was obtained from all subjects or, if subjects were under 18, from a parent and/or legal guardian. All subjects were informed in advance about the purpose, tasks, foreseeable risks or discomforts, benefits, confidentiality, expected duration, and researchers’ contact information. All members of the research team obtained ethics certification from the National Institutes of Health (NIH). All experimental protocols were subject to approval by the COBE Human Subjects Committee.

## Supplementary information

Appendix
