# Supplementary material for: Human decision-making biases in the moral dilemmas of autonomous vehicles
Source: Sci Rep. 2019 Sep 11;9:13080. doi: 10.1038/s41598-019-49411-7 (PMC6739396; doi:10.1038/s41598-019-49411-7)
Supplement: Supplementary file 1 — Appendix [file 41598_2019_49411_MOESM1_ESM.docx]

**Human decision-making biases in the moral dilemmas of autonomous vehicles**

Darius-Aurel Frank^1*^

Polymeros Chrysochou^1^

Panagiotis Mitkidis^1,2^

Dan Ariely^2^

^1^Department of Management, Aarhus University

^2^Center for Advanced Hindsight, Duke University

*Corresponding Author

Darius-Aurel Frank, PhD Candidate
Department of Management, Aarhus University,
Fuglesangs Allé 4, 8210 Aarhus V, Denmark

E-Mail: [df@mgmt.au.dk](mailto:df@mgmt.au.dk)

Supplementary materials
Figure 1. Visual elements for familiarizing participants in Studies 1–3, 5 and 6


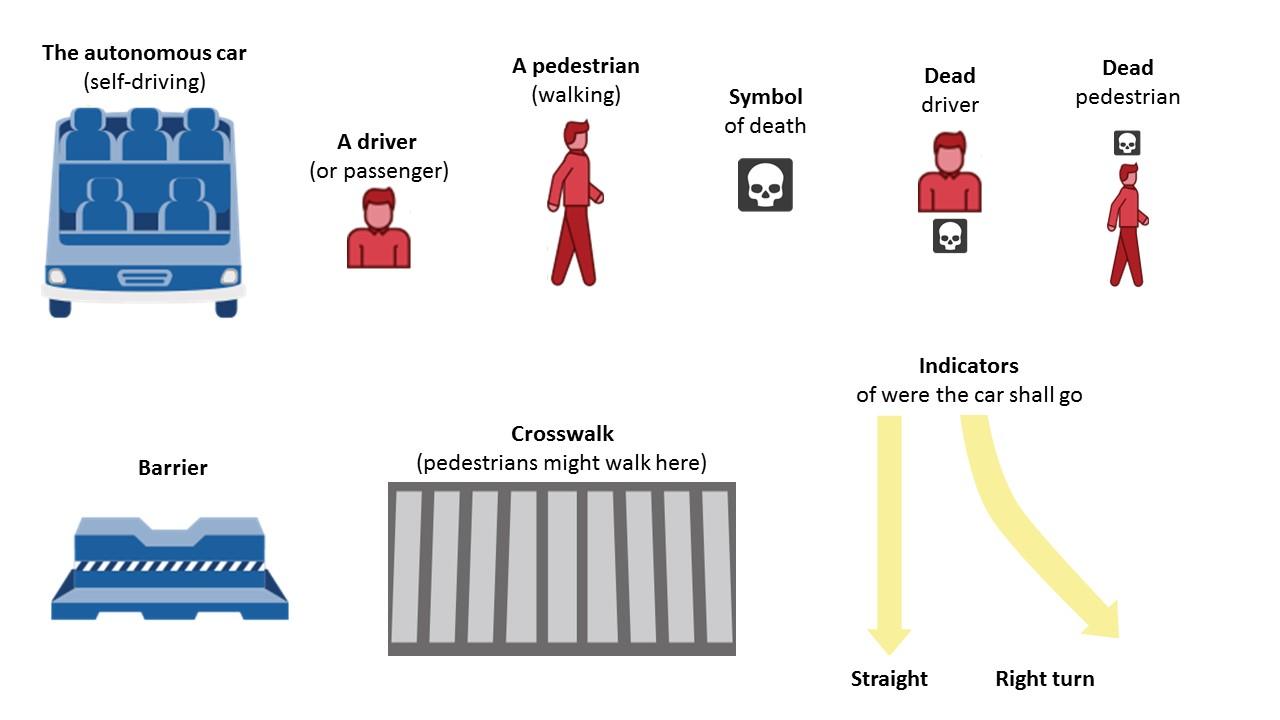


Note: Own work. Image assets adapted from the Moral Machine (<http://moralmachine.mit.edu/>) by Scalable Cooperation and MIT Media Lab [CC BY 4.0 (<https://creativecommons.org/licenses/by/4.0/>)].

*Figure* 2. Visual aid for instruction of passenger perspective in Studies 1, 2 and 7


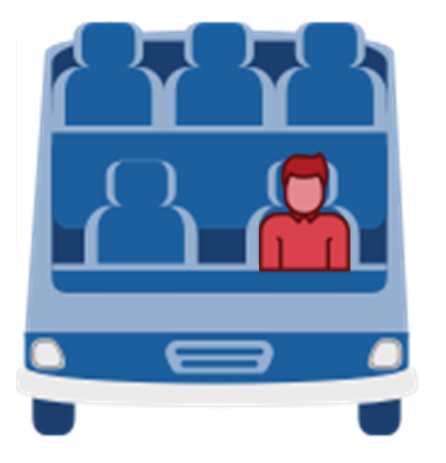


Note: Own work. Image assets adapted from the Moral Machine (<http://moralmachine.mit.edu/>) by Scalable Cooperation and MIT Media Lab [CC BY 4.0 (<https://creativecommons.org/licenses/by/4.0/>)].

*Figure* 3. Visual aid for instruction of pedestrian perspective in Studies 1–5 and 7


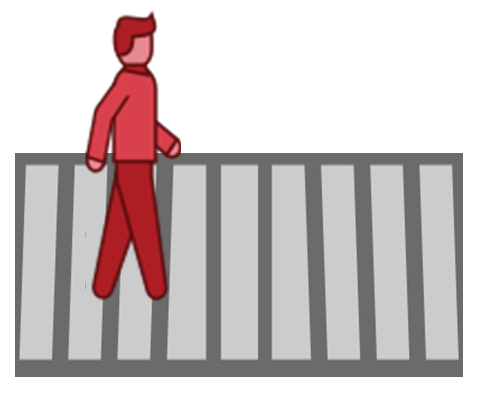


Note: Own work. Image assets adapted from the Moral Machine (<http://moralmachine.mit.edu/>) by Scalable Cooperation and MIT Media Lab [CC BY 4.0 (<https://creativecommons.org/licenses/by/4.0/>)].

# Pilot study of response time in Study 1

We pretested response times in a small pilot study (*N* = 26) using a span of five seconds in the decision-making task. The results showed that participants on average exceeded the time-limit by 2.91 seconds (*M*= 7.91, *SD* = 4.33). At the same time, participants reported good understanding of the dilemma (*M*= 5.54, *SD* = 2.04; 7-point bi-polar scale, 1 = “not at all”, 7 = “a great deal”). Based on this result, we decided to continue to use the instruction for five seconds in the intuitive decision-making mode condition to induce time-pressure.

# Robustness analyses of response time filtering in Study 1

We performed a series of analyses to support our decision to use a cutoff of seven seconds to separate intuitive from deliberate decisions. First, we performed a floodlight analysis to test for a possible interaction of response time and decision-making mode. As shown in Figure 4, we see a clear trend towards a significant interaction in the lower range of response times. In the range of observations, the interaction of decision-making mode and participants’ response time is not significant, t(803) = -0.65, p = 0.52.

*Figure* 4. Floodlight analysis of response time and decision-making mode in Study 1


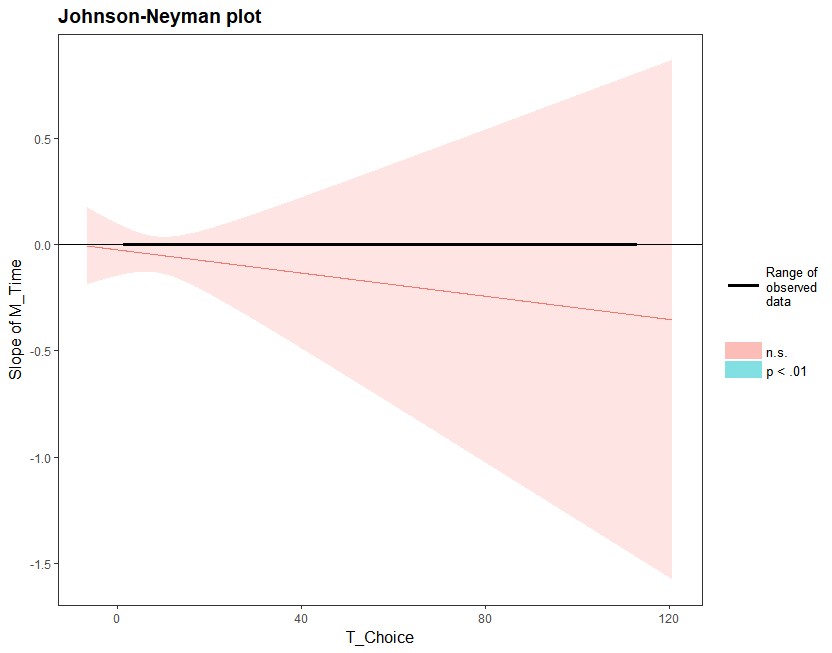


Next, we performed a correspondence analysis to probe the association of participants’ responses with the two decision-making modes at specific levels of response times (in seconds). Table 1 shows that the correspondence with intuitive decision-making mode shifts towards deliberate decision-making mode at around 7 seconds.

*Table* 1. Crosstabulation of response time and decision-making mode in Study 1

|  | Response time | | | | | | | | | | | | | | |  |
| --- | --- | --- | --- | --- | --- | --- | --- | --- | --- | --- | --- | --- | --- | --- | --- | --- |
| Decision-making mode | 1 | 2 | 3 | 4 | 5 | 6 | 7 | 8 | 9 | 10 | 11 | 12 | 13 | 14 | 15 | Total |
| Deliberate | 2 | 3 | 12 | 21 | 31 | 39 | 46 | 36 | 37 | 23 | 24 | 15 | 12 | 11 | 9 | 321 |
| Intuitive | 1 | 22 | 67 | 99 | 73 | 54 | 31 | 16 | 6 | 7 | 8 | 4 | 2 | 1 | 3 | 394 |

Note: Response time limited to 15 seconds for clarity.

Table 2 shows the same pattern for the distribution of responses across the two decision-making mode conditions.

*Table* 2. Column profiles of response time and decision-making mode in Study 1

|  | Response time | | | | | | | | | | | | | | |  |
| --- | --- | --- | --- | --- | --- | --- | --- | --- | --- | --- | --- | --- | --- | --- | --- | --- |
| Decision-making mode | 1 | 2 | 3 | 4 | 5 | 6 | 7 | 8 | 9 | 10 | 11 | 12 | 13 | 14 | 15 | Mass |
| Deliberate | 0.67 | 0.12 | 0.15 | 0.18 | 0.30 | 0.42 | 0.60 | 0.69 | 0.86 | 0.77 | 0.75 | 0.79 | 0.86 | 0.92 | 0.75 | 0.45 |
| Intuitive | 0.33 | 0.88 | 0.85 | 0.83 | 0.70 | 0.58 | 0.40 | 0.31 | 0.14 | 0.23 | 0.25 | 0.21 | 0.14 | 0.08 | 0.25 | 0.55 |

Note: Response time limited to 15 seconds for clarity.

# Manipulation check in Study 1

This limit resulted in a significant difference in participants’ response times between the two decision-making modes. Average response time (in seconds) is significantly lower in the intuitive (*M*= 4.71, *SD* = 1.15; *n* = 316) than in the deliberate decision-making condition (*M*= 14.16, *SD* = 9.00; *n* = 293), *t*(607) = -18.49, *p*< .001. Self-reported comprehension of the dilemma is high in both intuitive (*M*= 5.80, *SD* = 1.54) and deliberate (*M*= 6.46, *SD* = 1.01) decision-making condition but significantly different, t (607) = -6.28, *p*< 0.001. Self-reported confidence in the decision is significantly lower in deliberate (*M*= 4.57, *SD* = 2.02) than in intuitive decision-making (*M*= 5.11, *SD* = 1.95), *t*(607) = 3.32, *p*< .001.

*Figure* 5. Visual aid for instruction of passenger perspective in Study 3


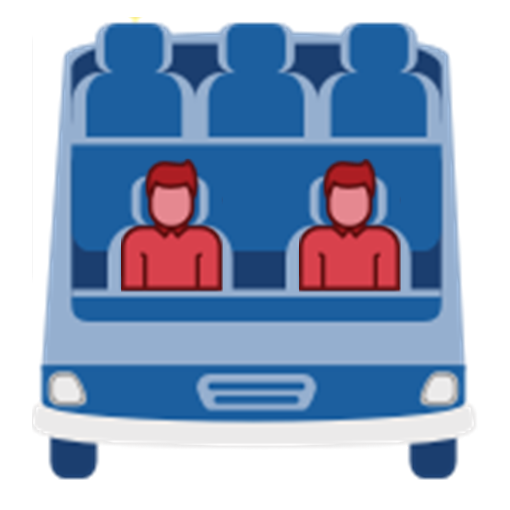


Note: Own work. Image assets adapted from the Moral Machine (<http://moralmachine.mit.edu/>) by Scalable Cooperation and MIT Media Lab [CC BY 4.0 (<https://creativecommons.org/licenses/by/4.0/>)].

*Figure* 6. Visual elements for familiarizing participants in Study 4


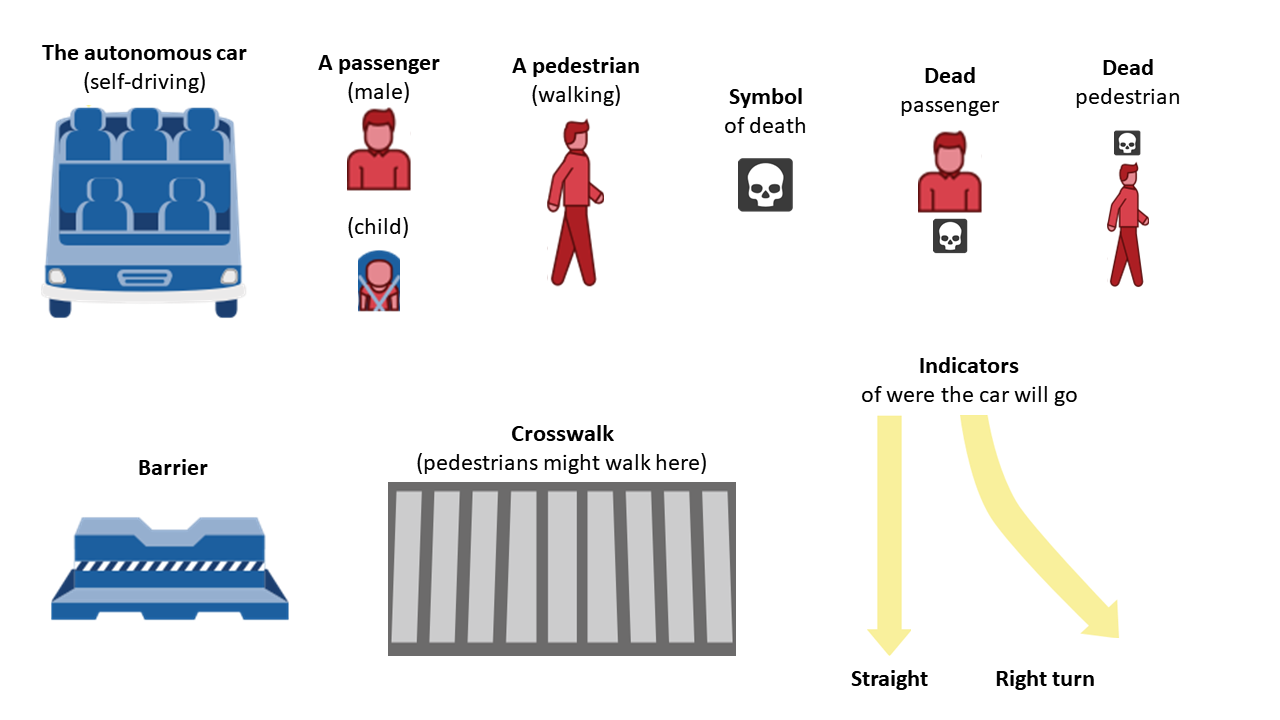


Note: Own work. Image assets adapted from the Moral Machine (<http://moralmachine.mit.edu/>) by Scalable Cooperation and MIT Media Lab [CC BY 4.0 (<https://creativecommons.org/licenses/by/4.0/>)].

*Figure* 7. Visual aid for instruction of passenger perspective in Study 4


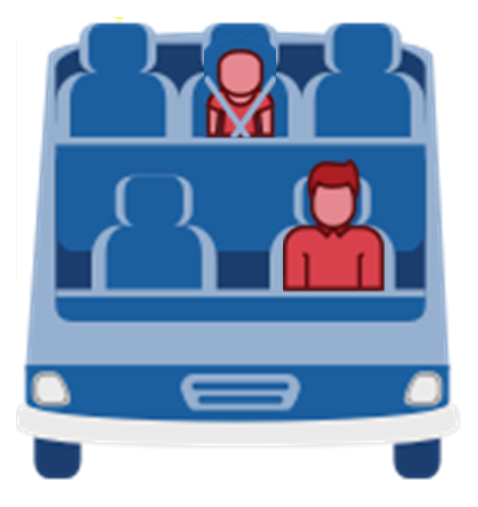


Note: Own work. Image assets adapted from the Moral Machine (<http://moralmachine.mit.edu/>) by Scalable Cooperation and MIT Media Lab [CC BY 4.0 (<https://creativecommons.org/licenses/by/4.0/>)].

*Figure* 8. Visual aid for instruction of passenger perspective in back-seat condition of Study 4


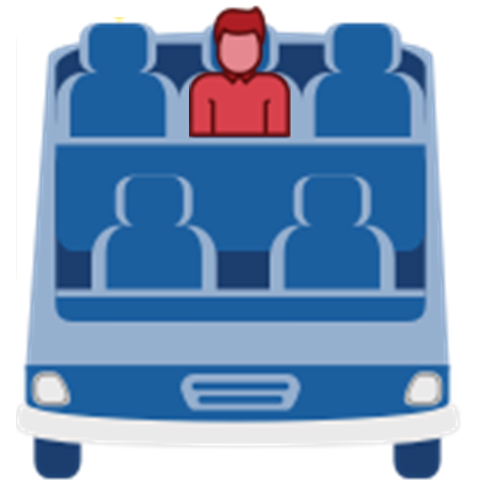


Note: Own work. Image assets adapted from the Moral Machine (<http://moralmachine.mit.edu/>) by Scalable Cooperation and MIT Media Lab [CC BY 4.0 (<https://creativecommons.org/licenses/by/4.0/>)].

*Figure* 9. Visual aid for instruction of passenger perspective in front-seat condition of Study 4


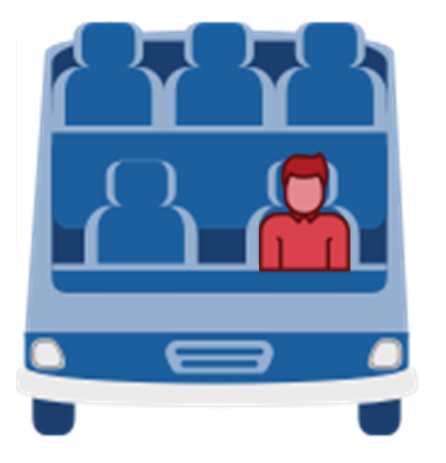


Note: Own work. Image assets adapted from the Moral Machine (<http://moralmachine.mit.edu/>) by Scalable Cooperation and MIT Media Lab [CC BY 4.0 (<https://creativecommons.org/licenses/by/4.0/>)].

*Figure* 10. Visual aid for instruction of pedestrian perspective in control condition of Study 6


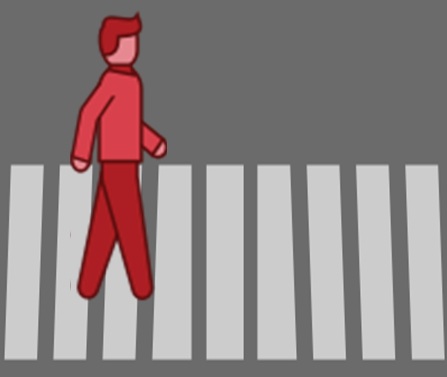


Note: Own work. Image assets adapted from the Moral Machine (<http://moralmachine.mit.edu/>) by Scalable Cooperation and MIT Media Lab [CC BY 4.0 (<https://creativecommons.org/licenses/by/4.0/>)].

*Figure* 11. Visual aid for instruction of pedestrian perspective in low norm violation condition of Study 6


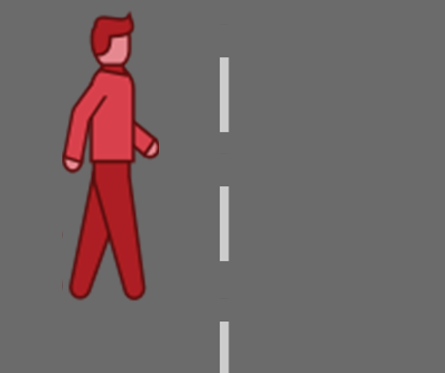


Note: Own work. Image assets adapted from the Moral Machine (<http://moralmachine.mit.edu/>) by Scalable Cooperation and MIT Media Lab [CC BY 4.0 (<https://creativecommons.org/licenses/by/4.0/>)].

*Figure* 12. Visual aid for instruction of pedestrian perspective in high norm violation condition of Study 6


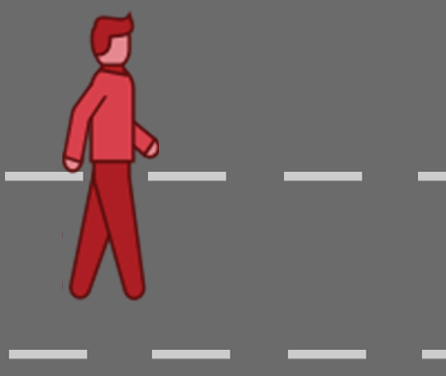


Note: Own work. Image assets adapted from the Moral Machine (<http://moralmachine.mit.edu/>) by Scalable Cooperation and MIT Media Lab [CC BY 4.0 (<https://creativecommons.org/licenses/by/4.0/>)].

*Figure* 13. Visual elements for familiarizing participants in Study 7
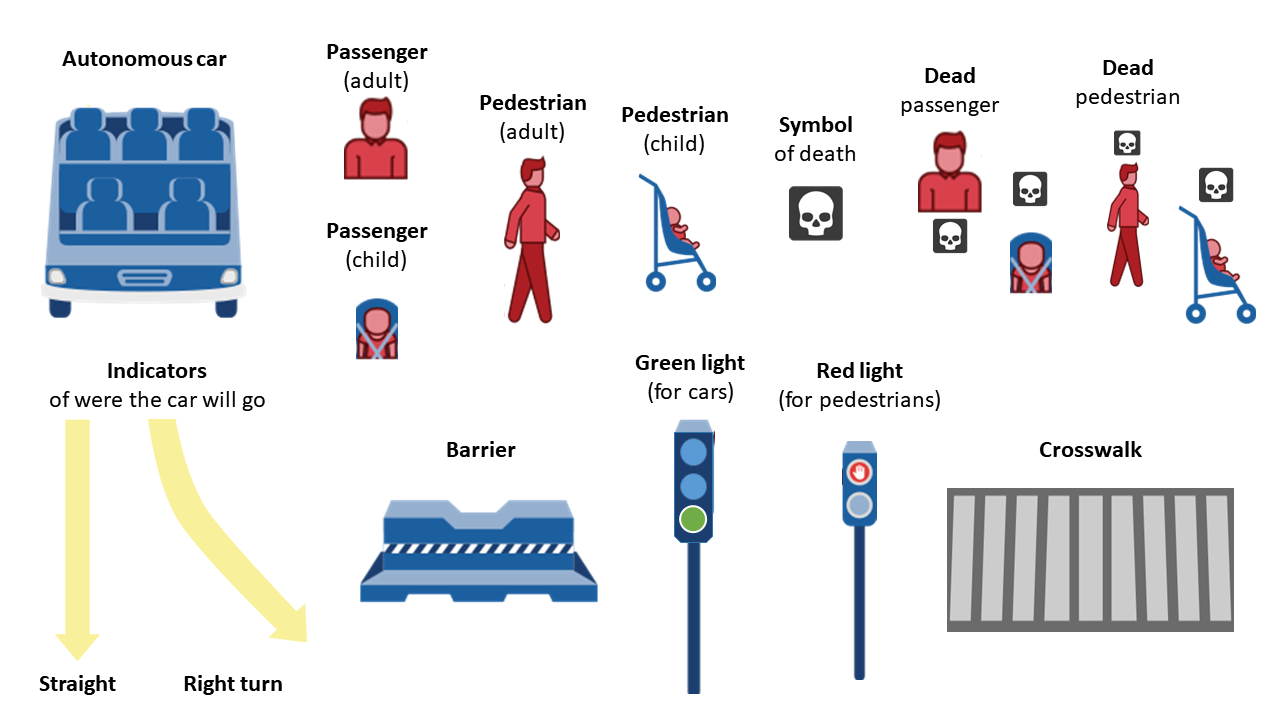


Note: Own work. Image assets adapted from the Moral Machine (<http://moralmachine.mit.edu/>) by Scalable Cooperation and MIT Media Lab [CC BY 4.0 (<https://creativecommons.org/licenses/by/4.0/>)].

*Figure* 14. The default path of the vehicle in dilemma 1 in Study 7


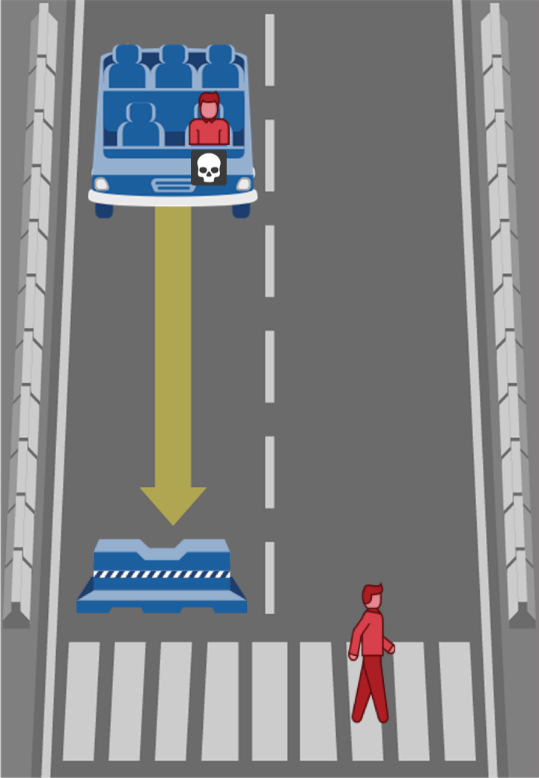


Note: Own work. Image assets adapted from the Moral Machine (<http://moralmachine.mit.edu/>) by Scalable Cooperation and MIT Media Lab [CC BY 4.0 (<https://creativecommons.org/licenses/by/4.0/>)].

*Figure* 15. The default path of the vehicle in dilemma 2 in Study 7


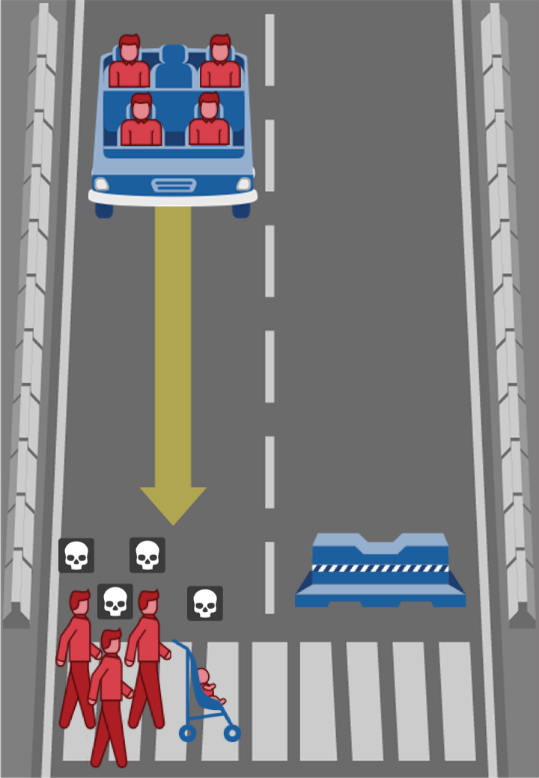


Note: Own work. Image assets adapted from the Moral Machine (<http://moralmachine.mit.edu/>) by Scalable Cooperation and MIT Media Lab [CC BY 4.0 (<https://creativecommons.org/licenses/by/4.0/>)].

*Figure* 16. The default path of the vehicle in dilemma 3 in Study 7


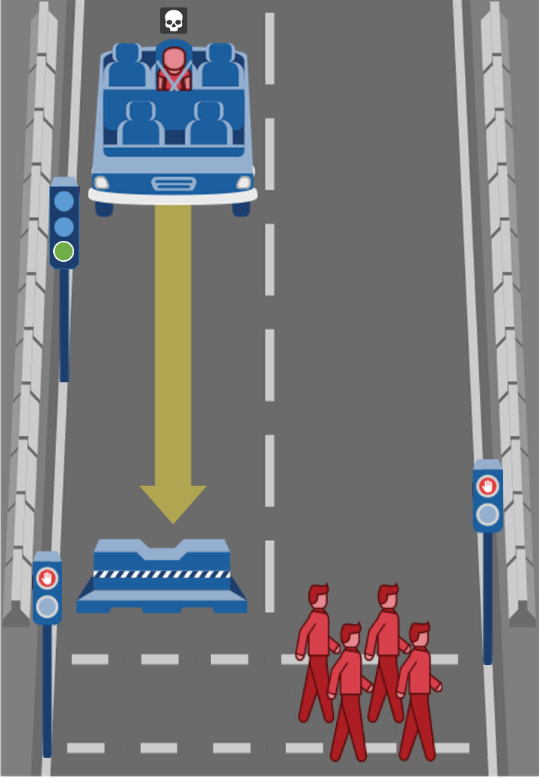


Note: Own work. Image assets adapted from the Moral Machine (<http://moralmachine.mit.edu/>) by Scalable Cooperation and MIT Media Lab [CC BY 4.0 (<https://creativecommons.org/licenses/by/4.0/>)].

*Figure* 17. The default path of the vehicle in dilemma 4 in Study 7


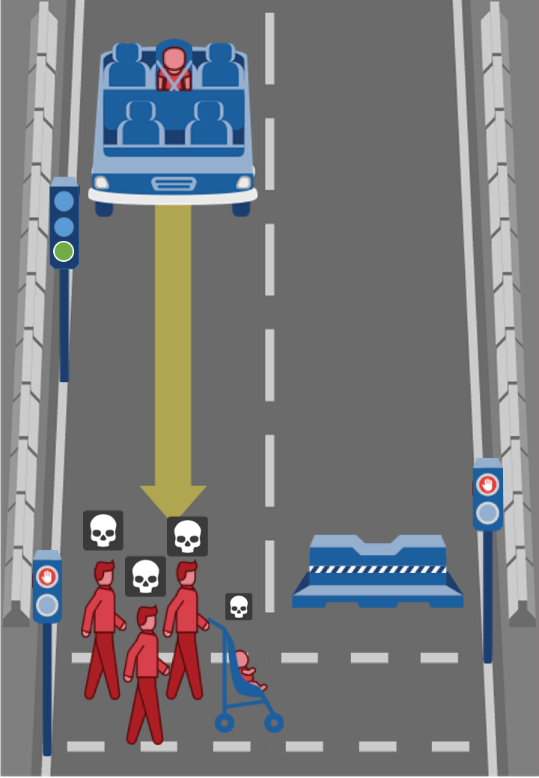


Note: Own work. Image assets adapted from the Moral Machine (<http://moralmachine.mit.edu/>) by Scalable Cooperation and MIT Media Lab [CC BY 4.0 (<https://creativecommons.org/licenses/by/4.0/>)].

*Figure* 18. The default path of the vehicle in dilemma 5 in Study 7


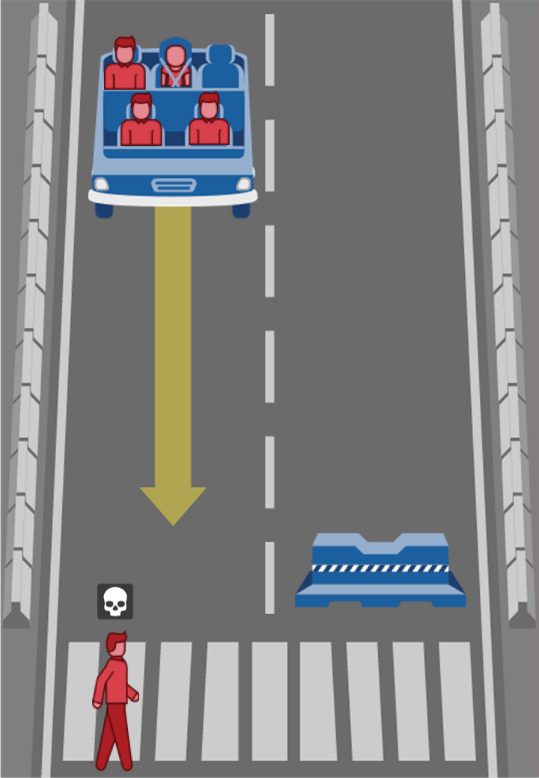


Note: Own work. Image assets adapted from the Moral Machine (<http://moralmachine.mit.edu/>) by Scalable Cooperation and MIT Media Lab [CC BY 4.0 (<https://creativecommons.org/licenses/by/4.0/>)].

*Figure* 19. The default path of the vehicle in dilemma 6 in Study 7


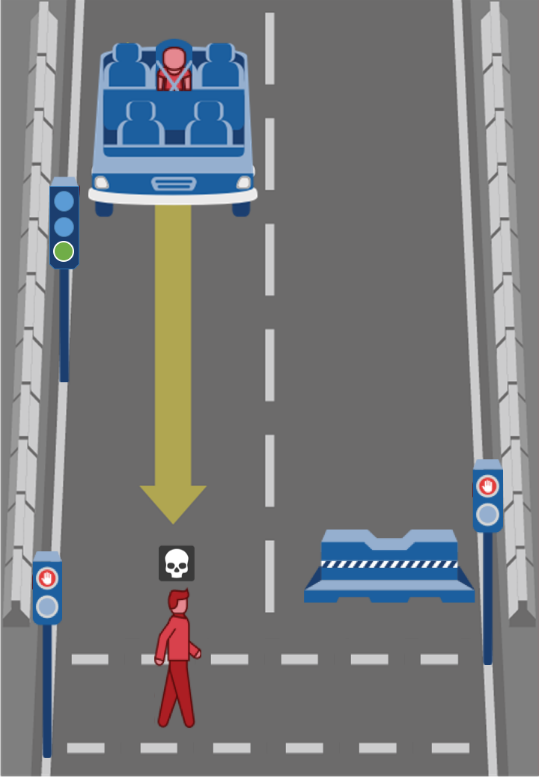


Note: Own work. Image assets adapted from the Moral Machine (<http://moralmachine.mit.edu/>) by Scalable Cooperation and MIT Media Lab [CC BY 4.0 (<https://creativecommons.org/licenses/by/4.0/>)].

*Figure* 20. The default path of the vehicle in dilemma 7 in Study 7


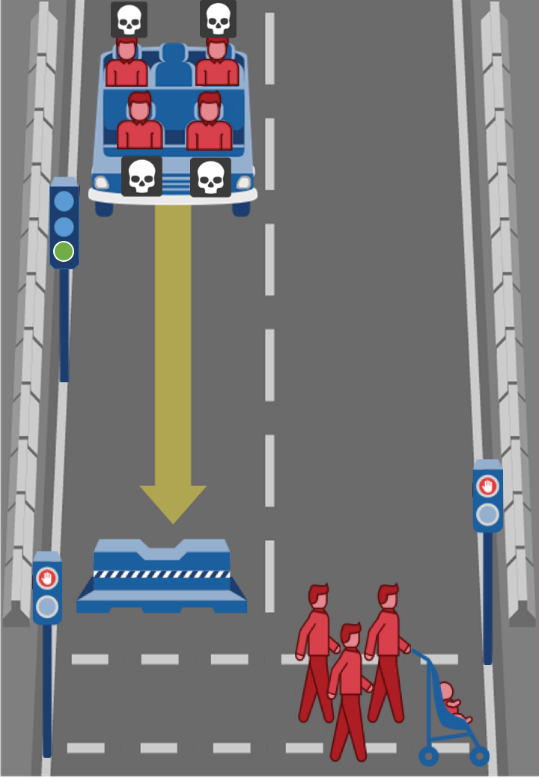


Note: Own work. Image assets adapted from the Moral Machine (<http://moralmachine.mit.edu/>) by Scalable Cooperation and MIT Media Lab [CC BY 4.0 (<https://creativecommons.org/licenses/by/4.0/>)].

*Figure* 21. The default path of the vehicle in dilemma 8 in Study 7


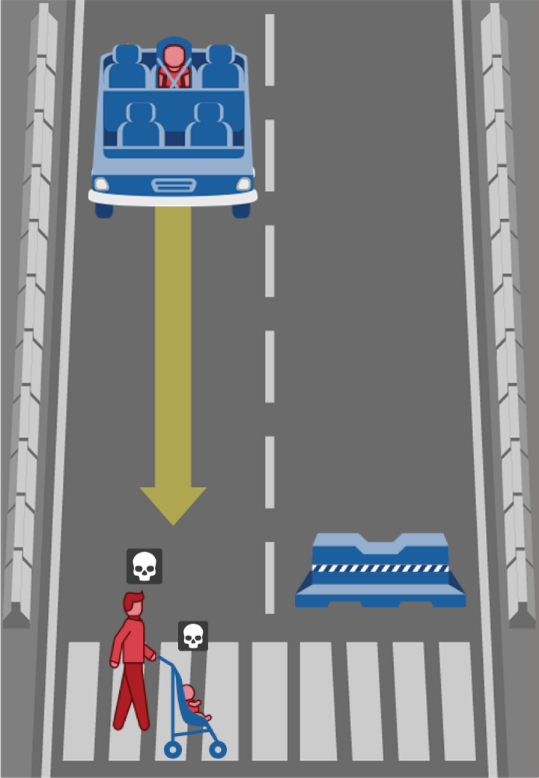


Note: Own work. Image assets adapted from the Moral Machine (<http://moralmachine.mit.edu/>) by Scalable Cooperation and MIT Media Lab [CC BY 4.0 (<https://creativecommons.org/licenses/by/4.0/>)].

*Figure* 22. The default path of the vehicle in dilemma 9 in Study 7


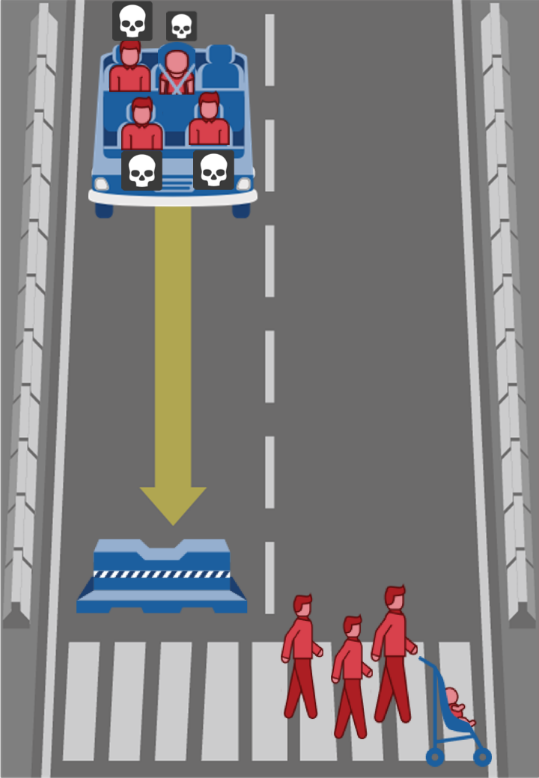


Note: Own work. Image assets adapted from the Moral Machine (<http://moralmachine.mit.edu/>) by Scalable Cooperation and MIT Media Lab [CC BY 4.0 (<https://creativecommons.org/licenses/by/4.0/>)].

*Figure* 23. The default path of the vehicle in dilemma 10 in Study 7


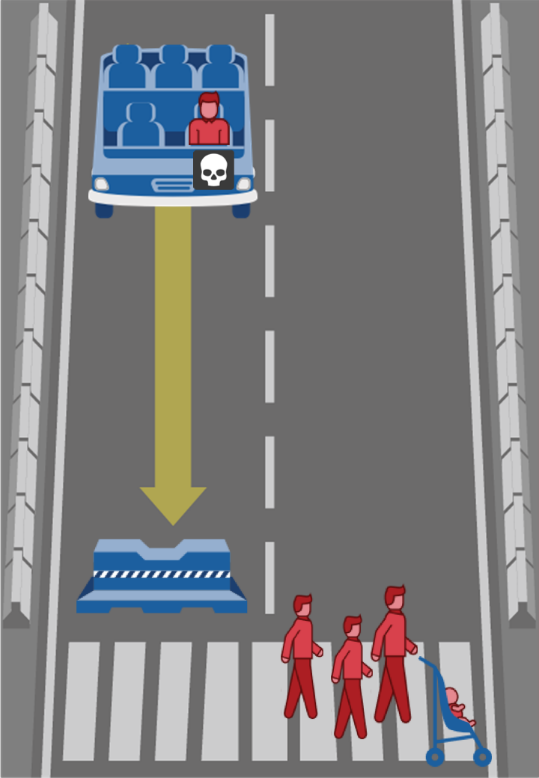


Note: Own work. Image assets adapted from the Moral Machine (<http://moralmachine.mit.edu/>) by Scalable Cooperation and MIT Media Lab [CC BY 4.0 (<https://creativecommons.org/licenses/by/4.0/>)].

*Figure* 24. The default path of the vehicle in dilemma 11 in Study 7


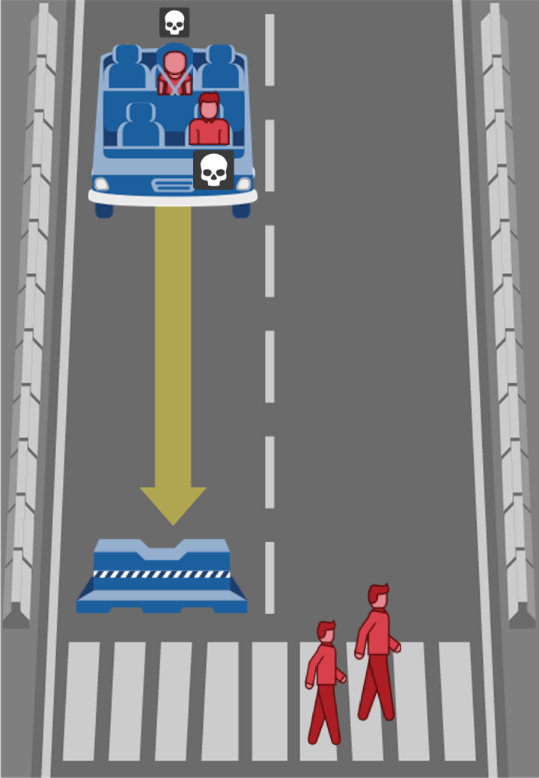


Note: Own work. Image assets adapted from the Moral Machine (<http://moralmachine.mit.edu/>) by Scalable Cooperation and MIT Media Lab [CC BY 4.0 (<https://creativecommons.org/licenses/by/4.0/>)].

*Figure* 25. The default path of the vehicle in dilemma 12 in Study 7


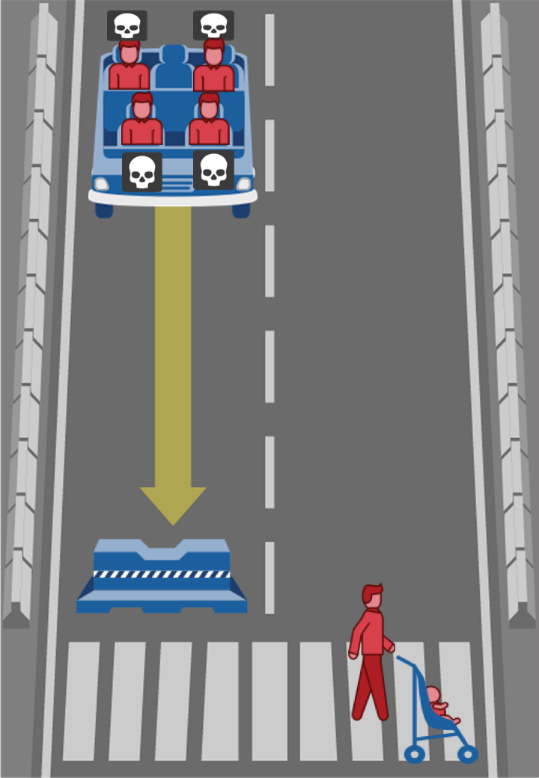


Note: Own work. Image assets adapted from the Moral Machine (<http://moralmachine.mit.edu/>) by Scalable Cooperation and MIT Media Lab [CC BY 4.0 (<https://creativecommons.org/licenses/by/4.0/>)].

*Figure* 26. The default path of the vehicle in dilemma 13 in Study 7


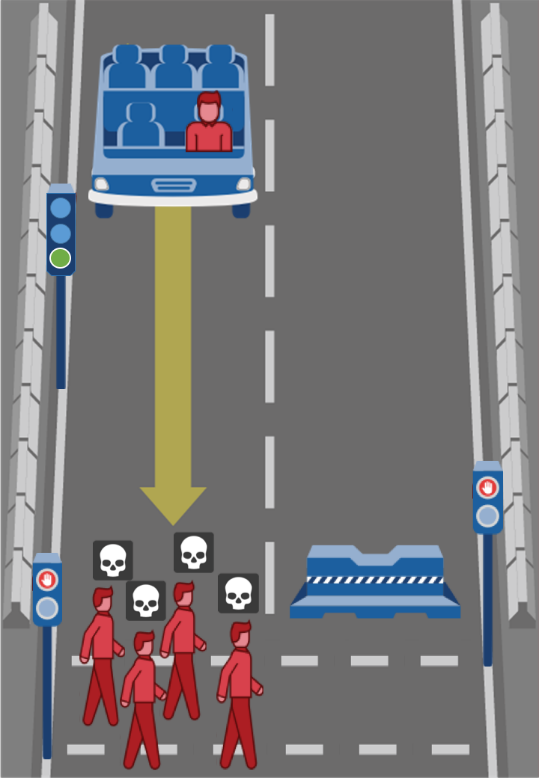


Note: Own work. Image assets adapted from the Moral Machine (<http://moralmachine.mit.edu/>) by Scalable Cooperation and MIT Media Lab [CC BY 4.0 (<https://creativecommons.org/licenses/by/4.0/>)].

*Figure* 27. The default path of the vehicle in dilemma 14 in Study 7


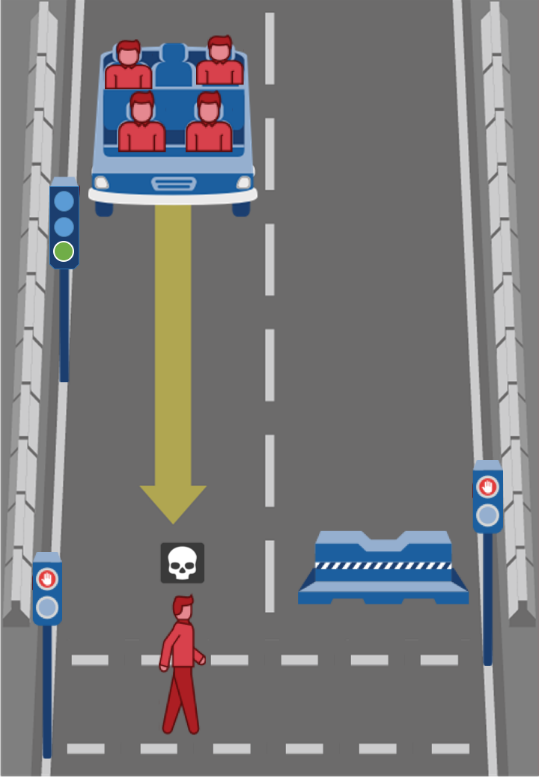


Note: Own work. Image assets adapted from the Moral Machine (<http://moralmachine.mit.edu/>) by Scalable Cooperation and MIT Media Lab [CC BY 4.0 (<https://creativecommons.org/licenses/by/4.0/>)].

*Figure* 28. The default path of the vehicle in dilemma 15 in Study 7


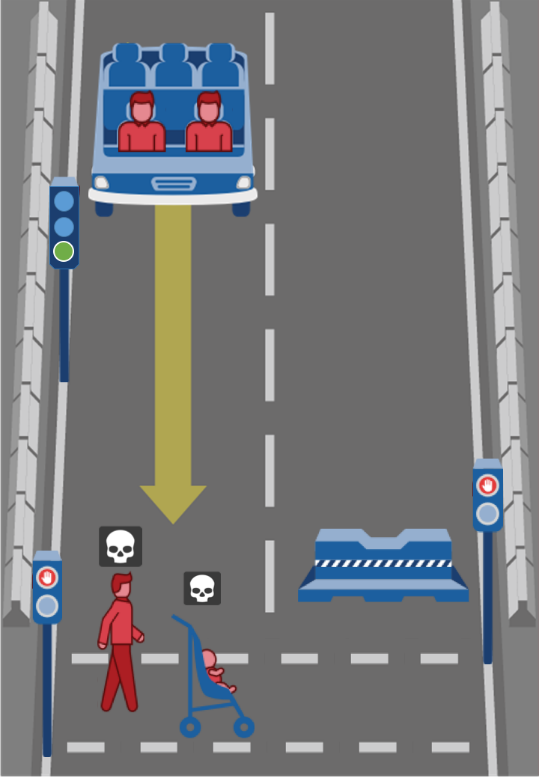


Note: Own work. Image assets adapted from the Moral Machine (<http://moralmachine.mit.edu/>) by Scalable Cooperation and MIT Media Lab [CC BY 4.0 (<https://creativecommons.org/licenses/by/4.0/>)].

*Figure* 29. The default path of the vehicle in dilemma 16 in Study 7


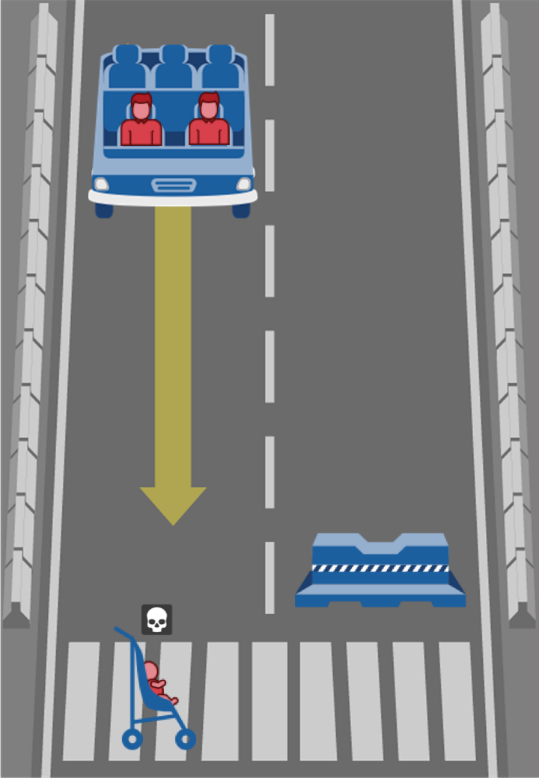


Note: Own work. Image assets adapted from the Moral Machine (<http://moralmachine.mit.edu/>) by Scalable Cooperation and MIT Media Lab [CC BY 4.0 (<https://creativecommons.org/licenses/by/4.0/>)].

*Figure* 30. The default path of the vehicle in dilemma 17 in Study 7


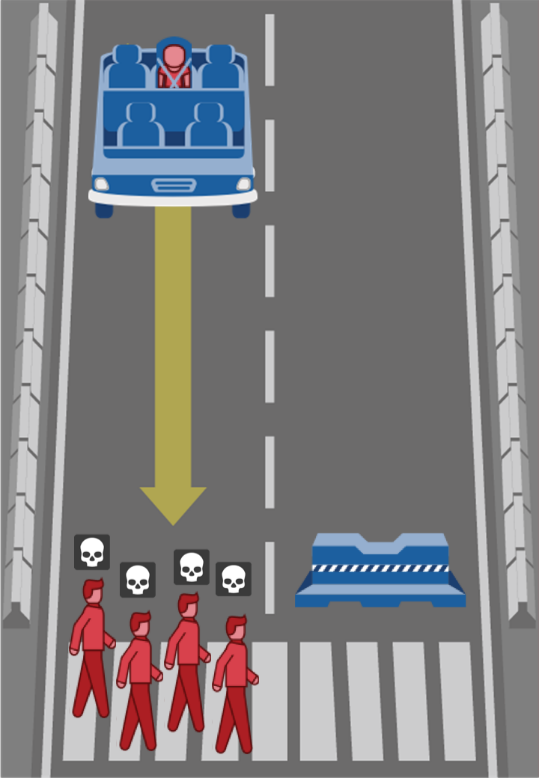


Note: Own work. Image assets adapted from the Moral Machine (<http://moralmachine.mit.edu/>) by Scalable Cooperation and MIT Media Lab [CC BY 4.0 (<https://creativecommons.org/licenses/by/4.0/>)].

*Figure* 31. The default path of the vehicle in dilemma 18 in Study 7


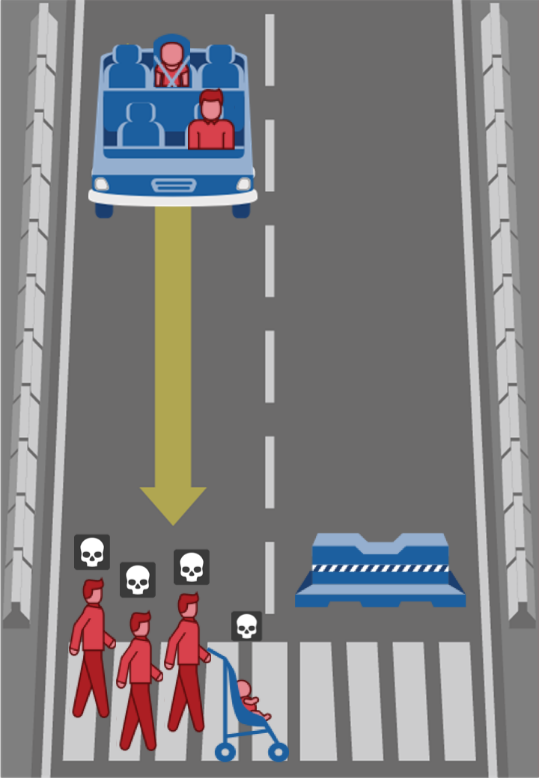


Note: Own work. Image assets adapted from the Moral Machine (<http://moralmachine.mit.edu/>) by Scalable Cooperation and MIT Media Lab [CC BY 4.0 (<https://creativecommons.org/licenses/by/4.0/>)].

*Figure* 32. The default path of the vehicle in dilemma 19 in Study 7


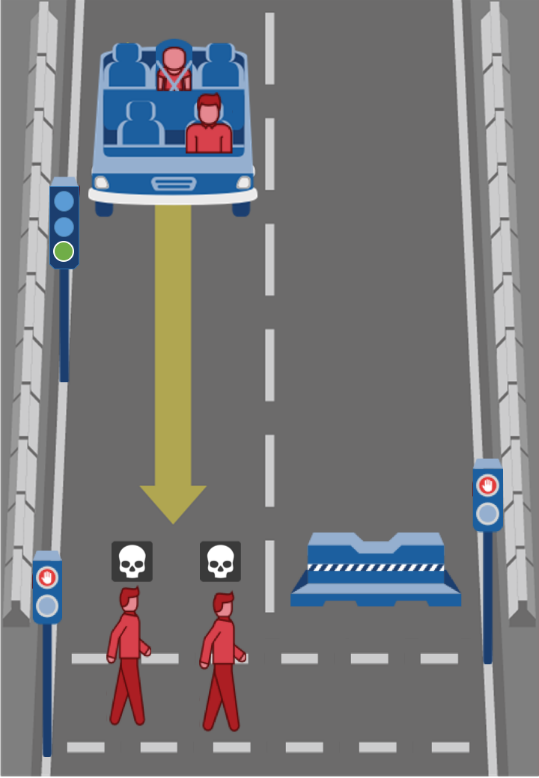


Note: Own work. Image assets adapted from the Moral Machine (<http://moralmachine.mit.edu/>) by Scalable Cooperation and MIT Media Lab [CC BY 4.0 (<https://creativecommons.org/licenses/by/4.0/>)].

*Figure* 33. The default path of the vehicle in dilemma 20 in Study 7


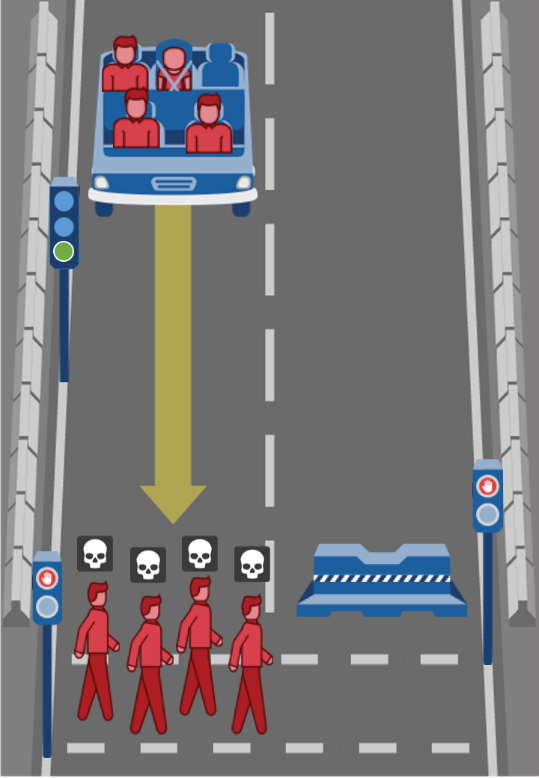


Note: Own work. Image assets adapted from the Moral Machine (<http://moralmachine.mit.edu/>) by Scalable Cooperation and MIT Media Lab [CC BY 4.0 (<https://creativecommons.org/licenses/by/4.0/>)].

*Figure* 34. The default path of the vehicle in dilemma 21 in Study 7


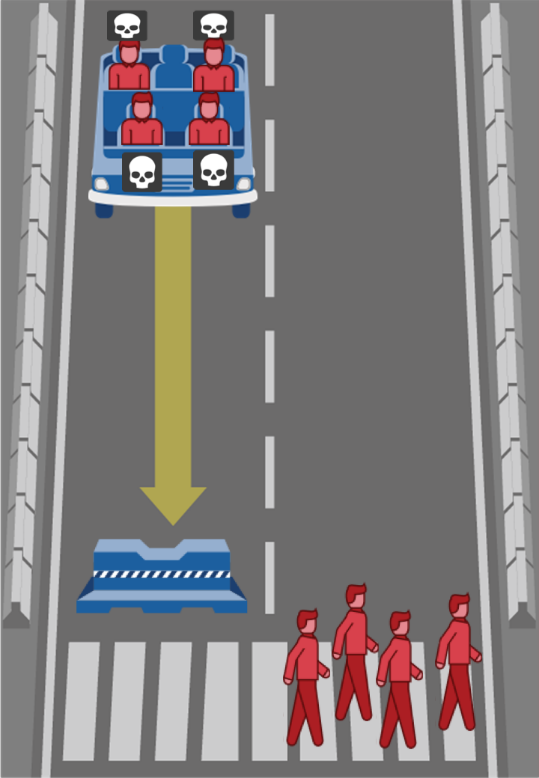


Note: Own work. Image assets adapted from the Moral Machine (<http://moralmachine.mit.edu/>) by Scalable Cooperation and MIT Media Lab [CC BY 4.0 (<https://creativecommons.org/licenses/by/4.0/>)].

*Figure* 35. The default path of the vehicle in dilemma 22 in Study 7


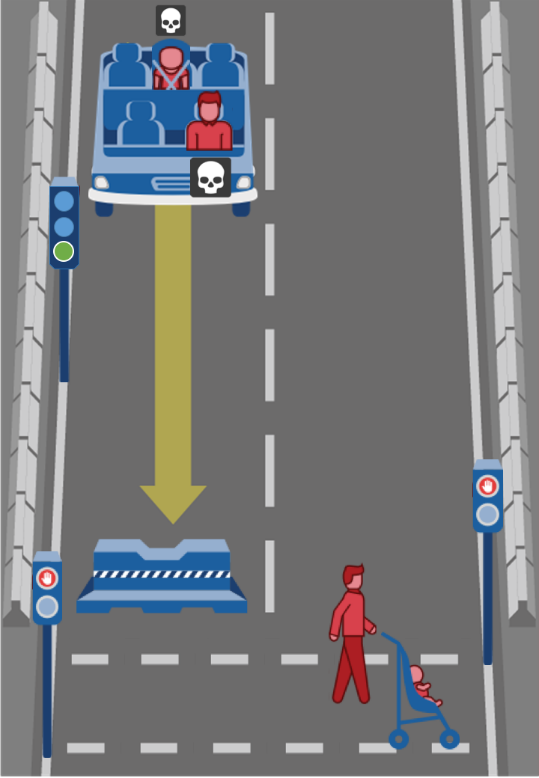


Note: Own work. Image assets adapted from the Moral Machine (<http://moralmachine.mit.edu/>) by Scalable Cooperation and MIT Media Lab [CC BY 4.0 (<https://creativecommons.org/licenses/by/4.0/>)].

*Figure* 36. The default path of the vehicle in dilemma 23 in Study 7


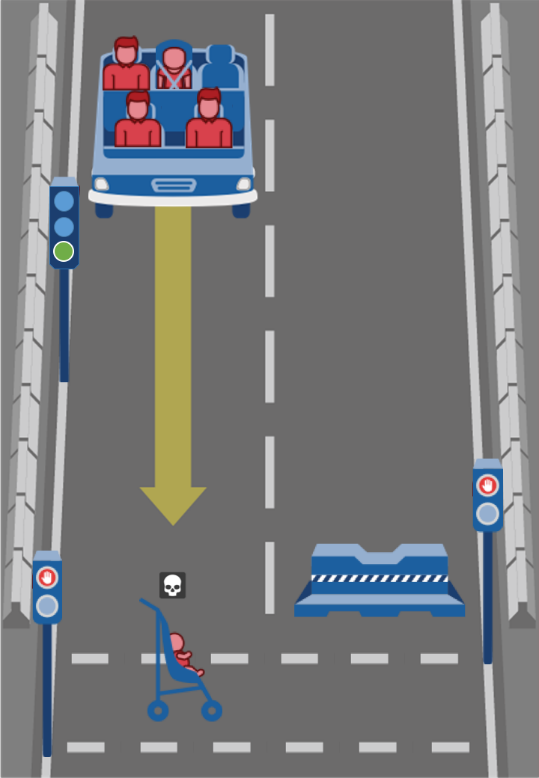


Note: Own work. Image assets adapted from the Moral Machine (<http://moralmachine.mit.edu/>) by Scalable Cooperation and MIT Media Lab [CC BY 4.0 (<https://creativecommons.org/licenses/by/4.0/>)].

*Figure* 37. The default path of the vehicle in dilemma 24 in Study 7


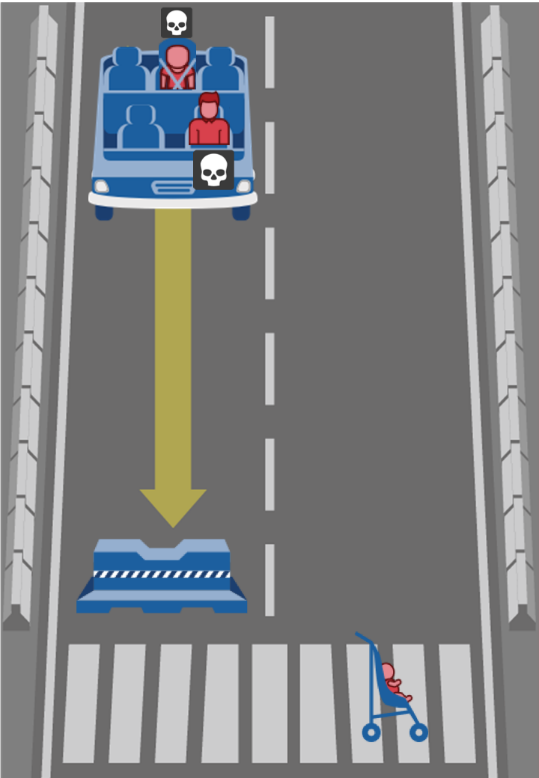


Note: Own work. Image assets adapted from the Moral Machine (<http://moralmachine.mit.edu/>) by Scalable Cooperation and MIT Media Lab [CC BY 4.0 (<https://creativecommons.org/licenses/by/4.0/>)].

*Figure* 38. The default path of the vehicle in dilemma 25 in Study 7


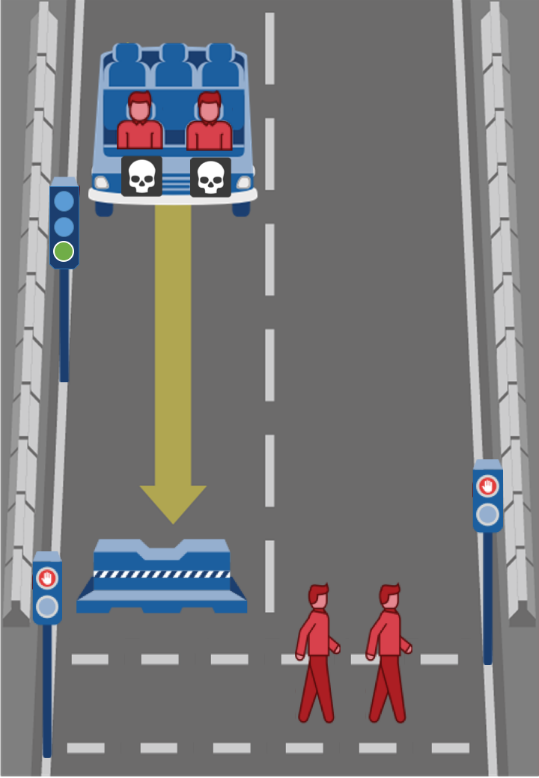


Note: Own work. Image assets adapted from the Moral Machine (<http://moralmachine.mit.edu/>) by Scalable Cooperation and MIT Media Lab [CC BY 4.0 (<https://creativecommons.org/licenses/by/4.0/>)].

*Figure* 39. The default path of the vehicle in dilemma 26 in Study 7


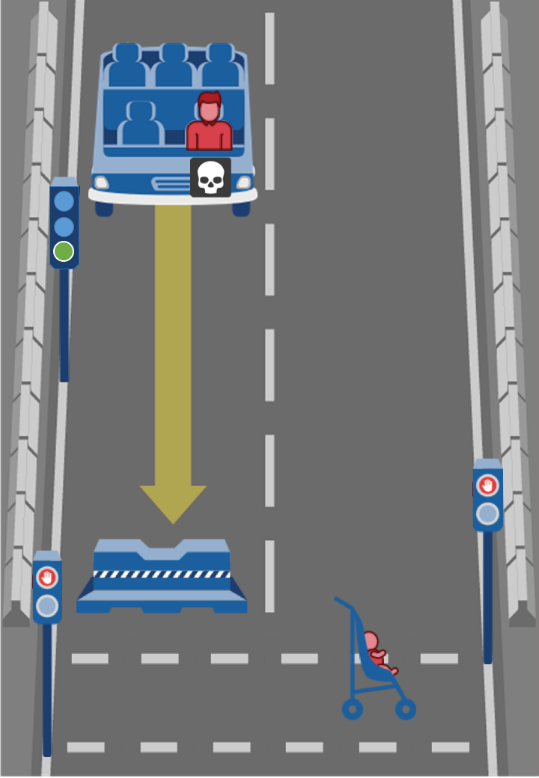


Note: Own work. Image assets adapted from the Moral Machine (<http://moralmachine.mit.edu/>) by Scalable Cooperation and MIT Media Lab [CC BY 4.0 (<https://creativecommons.org/licenses/by/4.0/>)].

*Figure* 40. The default path of the vehicle in dilemma 27 in Study 7


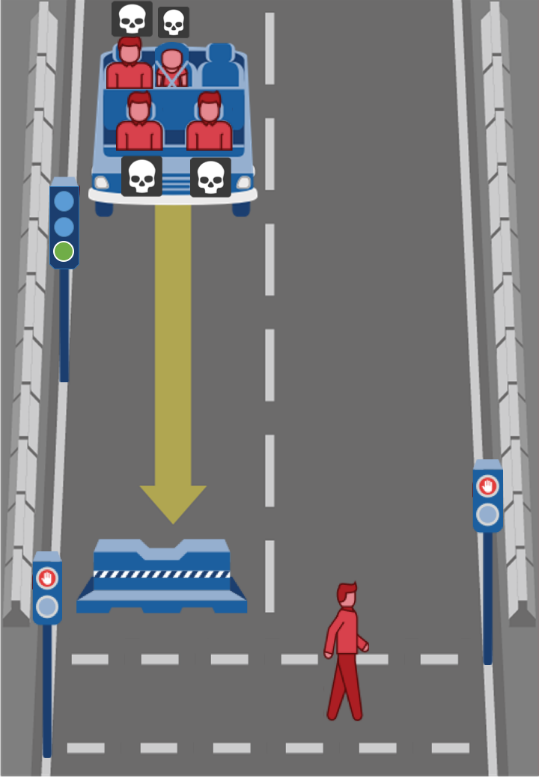


Note: Own work. Image assets adapted from the Moral Machine (<http://moralmachine.mit.edu/>) by Scalable Cooperation and MIT Media Lab [CC BY 4.0 (<https://creativecommons.org/licenses/by/4.0/>)].

*Figure* 41. The default path of the vehicle in dilemma 28 in Study 7


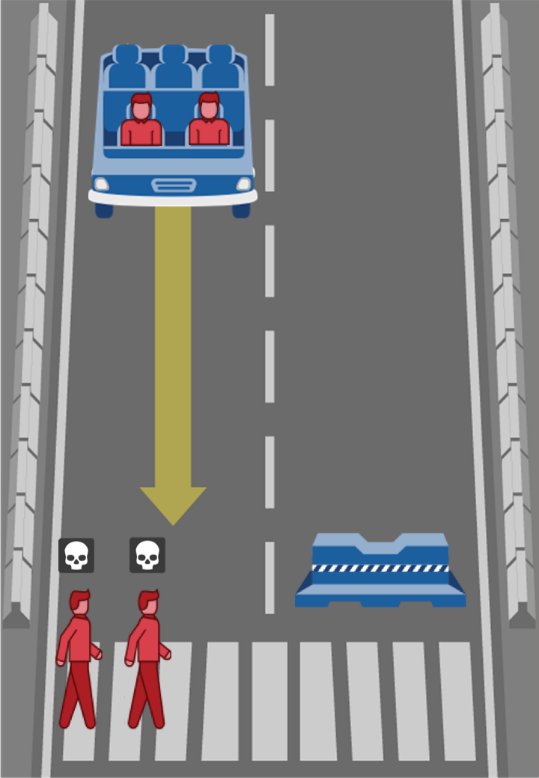


Note: Own work. Image assets adapted from the Moral Machine (<http://moralmachine.mit.edu/>) by Scalable Cooperation and MIT Media Lab [CC BY 4.0 (<https://creativecommons.org/licenses/by/4.0/>)].

# Prequestionnaire in Study 7

All text-based dilemmas were taken from the supplementary materials provided in Greene, J. D., Sommerville, R. B., Nystrom, L. E., Darley, J. M., & Cohen, J. D. (2001). An fMRI Investigation of Emotional Engagement in Moral Judgment. Science, 293(5537), 2105-2108. doi:10.1126/science.1062872.

The dilemmas were presented as shown in Table 3. Participants read each dilemma paragraph by paragraph and indicated their moral judgement by pressing “appropriate” or “inappropriate” after reading the final question for each text-based dilemma.

*Table* 3. Text-based dilemmas used in the prequestionnaire of Study 7

| Dilemma | Moral-impersonal | Moral-personal |
| --- | --- | --- |
| 1 | You are at the wheel of a runaway trolley quickly approaching a fork in the tracks. On the tracks extending to the left is a group of five railway workmen. On the tracks extending to the right is a single railway workman.  If you do nothing the trolley will proceed to the left, causing the deaths of the five workmen. The only way to avoid the deaths of these workmen is to hit a switch on your dashboard that will cause the trolley to proceed to the right, causing the death of the single workman.  Is it appropriate for you to hit the switch in order to avoid the deaths of the five workmen? | A runaway trolley is heading down the tracks toward five workmen who will be killed if the trolley proceeds on its present course. You are on a footbridge over the tracks, in between the approaching trolley and the five workmen. Next to you on this footbridge is a stranger who happens to be very large.  The only way to save the lives of the five workmen is to push this stranger off the bridge and onto the tracks below where his large body will stop the trolley. The stranger will die if you do this, but the five workmen will be saved.  Is it appropriate for you to push the stranger on to the tracks in order to save the five workmen? |
| 2 | You are at home one day when the mail arrives. You receive a letter from a reputable international aid organization. The letter asks you to make a donation of two hundred dollars to their organization.  The letter explains that a two hundred-dollar donation will allow this organization to provide needed medical attention to some poor people in another part of the world.  Is it appropriate for you to not make a donation to this organization in order to save money? | Your plane has crashed in the Himalayas. The only survivors are yourself, another man, and a young boy. The three of you travel for days, battling extreme cold and wind. Your only chance at survival is to find your way to small a village on the other side of the mountain, several days away.  The boy has a broken leg and cannot move very quickly. His chances of surviving the journey are essentially zero. Without food, you and the other man will probably die as well. The other man suggests that you sacrifice the boy and eat his remains over the next few days.  Is it appropriate to kill this boy so that you and the man may survive your journey to safety? |
| 3 | You are walking down the street when you come across a wallet lying on the ground. You open the wallet and find that it contains several hundred dollars in cash as well the owner's driver's license.  From the credit cards and other items in the wallet it's very clear that the wallet's owner is wealthy. You, on the other hand, have been hit by hard times recently and could really use some extra money. You consider sending the wallet back to the owner without the cash, keeping the cash for yourself.  Is it appropriate for you to keep the money you found in the wallet in order to have more money for yourself? | Enemy soldiers have taken over your village. They have orders to kill all remaining civilians. You and some of your townspeople have sought refuge in the cellar of a large house. Outside you hear the voices of soldiers who have come to search the house for valuables.  Your baby begins to cry loudly. You cover his mouth to block the sound. If you remove your hand from his mouth his crying will summon the attention of the soldiers who will kill you, your child, and the others hiding out in the cellar. To save yourself and the others you must smother your child to death.  Is it appropriate for you to smother your child in order to save yourself and the other townspeople? |

*Note*: The first moral-impersonal dilemma represents the original trolley problem. The first moral-personal dilemma represents the footbridge dilemma.

*Figure* 42. Moral judgements on text-based dilemmas in Study 7


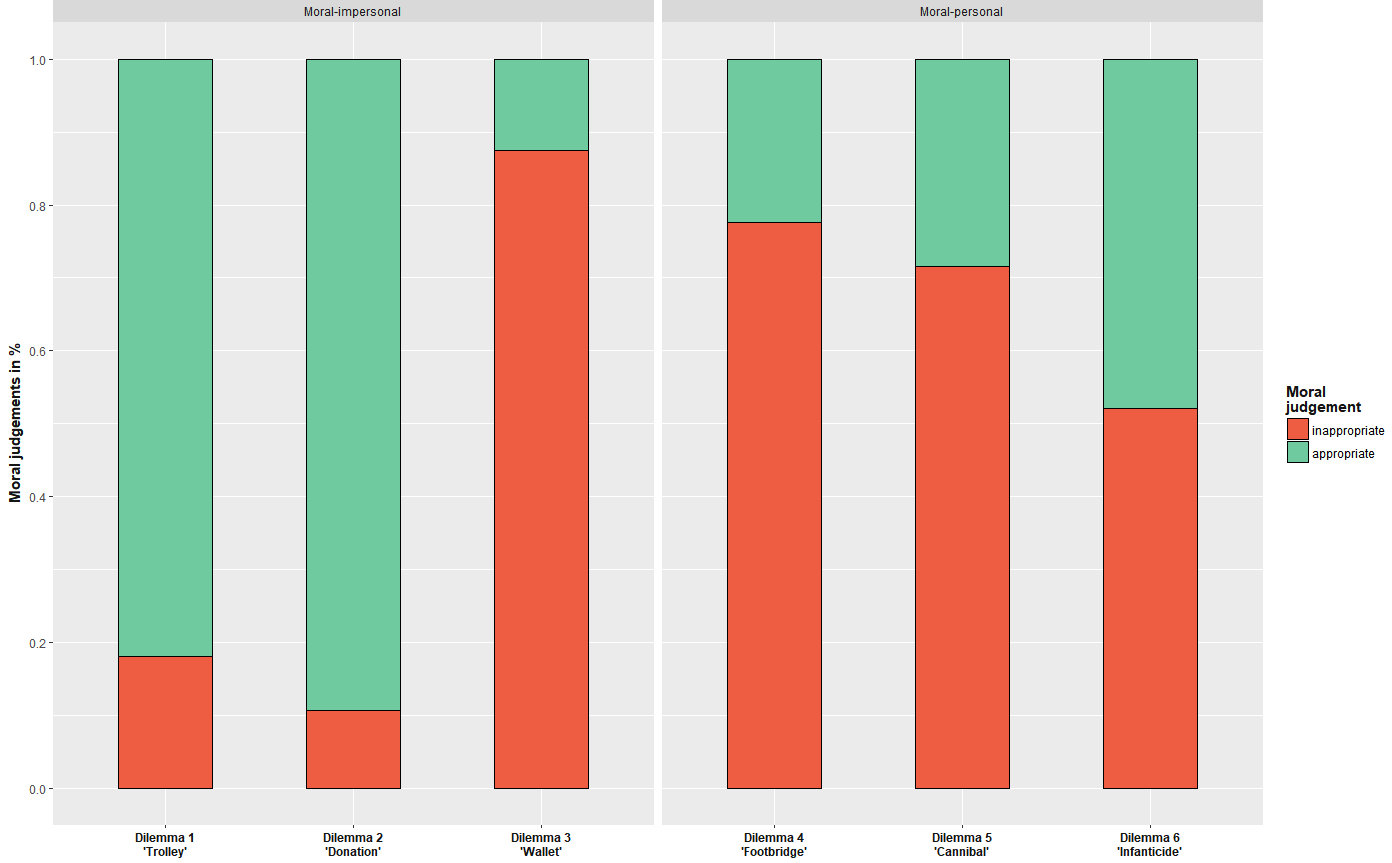


Note: The names of the dilemmas were not shown to participants.

*Table* 4. Factors, levels and number of combinations in Study 7

| Factor | Levels | Used in | Combinations | |
| --- | --- | --- | --- | --- |
|  |  |  | Max | Min |
| Number of Passengers | 1, 2, 4 | Study 3 | 216 | 16 |
| Number of Pedestrians | 1, 2, 4 |  |  |  |
| Kid among Pedestrians | Yes, No | Study 4 |  |  |
| Kid among Passengers | Yes, No |  |  |  |
| Unlawful Pedestrian | Yes, No | Study 7 |  |  |
| Intervention | Straight kills Passenger(s), Intervention kills Passenger(s) | Study 1, 2 |  |  |

Note: Number of combinations computed with SPSS Statistics.

*Table* 5. Overview of dilemmas and factor combinations in Study 7

| Dilemma | Number of Passengers | Number of Pedestrians | Kid among Passengers | Kid among Pedestrians | Intervention | Unlawful Pedestrian |
| --- | --- | --- | --- | --- | --- | --- |
| 1 | 1 | 1 | No | No | No | No |
| 2 | 4 | 4 | No | Yes | Yes | No |
| 3 | 1 | 4 | Yes | No | No | Yes |
| 4 | 1 | 4 | Yes | Yes | Yes | Yes |
| 5 | 4 | 1 | Yes | No | Yes | No |
| 6 | 1 | 1 | Yes | No | Yes | Yes |
| 7 | 4 | 4 | No | Yes | No | Yes |
| 8 | 1 | 2 | Yes | Yes | Yes | No |
| 9 | 4 | 4 | Yes | Yes | No | No |
| 10 | 1 | 4 | No | Yes | No | No |
| 11 | 2 | 2 | Yes | No | No | No |
| 12 | 4 | 2 | No | Yes | No | No |
| 13 | 1 | 4 | No | No | Yes | Yes |
| 14 | 4 | 1 | No | No | Yes | Yes |
| 15 | 2 | 2 | No | Yes | Yes | Yes |
| 16 | 2 | 1 | No | Yes | Yes | No |
| 17 | 1 | 4 | Yes | No | Yes | No |
| 18 | 2 | 4 | Yes | Yes | Yes | No |
| 19 | 2 | 2 | Yes | No | Yes | Yes |
| 20 | 4 | 4 | Yes | No | Yes | Yes |
| 21 | 4 | 4 | No | No | No | No |
| 22 | 2 | 2 | Yes | Yes | No | Yes |
| 23 | 4 | 1 | Yes | Yes | Yes | Yes |
| 24 | 2 | 1 | Yes | Yes | No | No |
| 25 | 2 | 2 | No | No | No | Yes |
| 26 | 1 | 1 | No | Yes | No | Yes |
| 27 | 4 | 1 | Yes | No | No | Yes |
| 28 | 2 | 2 | No | No | Yes | No |

*Table* 6. Summary data from Studies 1–6 for internal meta-analysis

| Study | Factor 1 | Factor 2 | *M* | *SD* | *n* |
| --- | --- | --- | --- | --- | --- |
| 1 | Deliberate | Observer | 0.38 | 0.49 | 97 |
| 1 | Deliberate | Pedestrian | 0.29 | 0.45 | 94 |
| 1 | Deliberate | Passenger | 0.42 | 0.50 | 102 |
| 1 | Intuitive | Observer | 0.24 | 0.43 | 105 |
| 1 | Intuitive | Pedestrian | 0.17 | 0.38 | 103 |
| 1 | Intuitive | Passenger | 0.23 | 0.42 | 108 |
| 2 | Deliberate | Observer | 0.29 | 0.46 | 110 |
| 2 | Deliberate | Pedestrian | 0.24 | 0.43 | 91 |
| 2 | Deliberate | Passenger | 0.41 | 0.49 | 104 |
| 2 | Intuitive | Observer | 0.22 | 0.42 | 98 |
| 2 | Intuitive | Pedestrian | 0.17 | 0.38 | 100 |
| 2 | Intuitive | Passenger | 0.22 | 0.42 | 100 |
| 3 | Deliberate | Observer | 0.42 | 0.50 | 45 |
| 3 | Deliberate | Pedestrian | 0.60 | 0.50 | 40 |
| 3 | Deliberate | Passenger | 0.50 | 0.51 | 42 |
| 3 | Intuitive | Observer | 0.26 | 0.45 | 53 |
| 3 | Intuitive | Pedestrian | 0.04 | 0.20 | 47 |
| 3 | Intuitive | Passenger | 0.29 | 0.46 | 59 |
| 4 | Deliberate | Observer | 0.57 | 0.50 | 47 |
| 4 | Deliberate | Pedestrian | 0.57 | 0.50 | 46 |
| 4 | Deliberate | Passenger | 0.50 | 0.50 | 52 |
| 4 | Intuitive | Observer | 0.34 | 0.48 | 50 |
| 4 | Intuitive | Pedestrian | 0.12 | 0.32 | 52 |
| 4 | Intuitive | Passenger | 0.34 | 0.48 | 61 |
| 5 | Deliberate | Observer | 0.27 | 0.45 | 103 |
| 5 | Deliberate | Pedestrian | 0.23 | 0.42 | 101 |
| 5 | Deliberate | Passenger | 0.32 | 0.47 | 98 |
| 5 | Intuitive | Observer | . | . | . |
| 5 | Intuitive | Pedestrian | . | . | . |
| 5 | Intuitive | Passenger | . | . | . |
| 6 | Deliberate | Observer | 0.33 | 0.47 | 119 |
| 6 | Deliberate | Pedestrian | 0.25 | 0.43 | 118 |
| 6 | Deliberate | Passenger | 0.28 | 0.45 | 116 |
| 6 | Intuitive | Observer | . | . | . |
| 6 | Intuitive | Pedestrian | . | . | . |
| 6 | Intuitive | Passenger | . | . | . |

*Table* 7. Contrasts used for internal meta-analysis in Study 7

| Contrast | Coding | Factor 1 | Factor 2 |
| --- | --- | --- | --- |
| 1 | -1 -1 -1 1 1 1 | Decision-making mode Intuitive vs. Deliberate |  |
| 2 | 0 -1 1 0 -1 1 |  | Perspective of Passenger vs. Pedestrian |
| 3 | -1 0 1 -1 0 1 |  | Perspective of Passenger vs. Observer |
| 4 | -1 1 0 -1 1 0 |  | Perspective of Pedestrian vs. Observer |
| 5 | 0 -1 1 0 0 0 | Deliberate decision-making mode | Perspective of Passenger vs. Pedestrian |
| 6 | -1 0 1 0 0 0 | Deliberate decision-making mode | Perspective of Passenger vs. Observer |
| 7 | -1 1 0 0 0 0 | Deliberate decision-making mode | Perspective of Pedestrian vs. Observer |
| 8 | 0 0 0 0 -1 1 | Intuitive decision-making mode | Perspective of Passenger vs. Pedestrian |
| 9 | 0 0 0 -1 0 1 | Intuitive decision-making mode | Perspective of Passenger vs. Observer |
| 10 | 0 0 0 -1 1 0 | Intuitive decision-making mode | Perspective of Pedestrian vs. Observer |

Note: Factor 1 refers to the condition of decision-making mode. Factor 2 refers to the condition of personal perspectives.

*Table* 8. Sample size requirements for contrasts of internal meta-analysis

| Contrast | *N* |
| --- | --- |
| 1 | 30 |
| 2 | 246 |
| 3 | *Inf* |
| 4 | 509 |
| 5 | *Inf* |
| 6 | *Inf* |
| 7 | *Inf* |
| 8 | 268 |
| 9 | *Inf* |
| 10 | 296 |

Note: This table states the sample size per condition required to achieve 80% power for each contrast. Inf means that 80% power cannot be achieved.

*Table* 9. Contrast estimated covariance matrix for main effects of internal meta-analysis

| Contrast | 1 | 2 | 2 | 4 |
| --- | --- | --- | --- | --- |
| 1 | 0.0079 | 0.0002 | -0.0001 | -0.0003 |
| 2 | 0.0002 | 0.0042 | 0.0023 | -0.0019 |
| 3 | -0.0001 | 0.0023 | 0.0046 | 0.0023 |
| 4 | -0.0003 | -0.0019 | 0.0023 | 0.0042 |

*Table* 10. Contrast estimated covariance matrix for simple effects of internal meta-analysis

| Contrast | 5 | 6 | 7 | 8 | 9 | 10 |
| --- | --- | --- | --- | --- | --- | --- |
| 5 | 0.0019 | 0.0009 | -0.0009 | -0.0000 | 0.0000 | 0.0000 |
| 6 | 0.0009 | 0.0019 | 0.0009 | 0.0000 | 0.0000 | 0.0000 |
| 7 | -0.0009 | 0.0009 | 0.0018 | 0.0000 | -0.0000 | -0.0000 |
| 8 | -0.0000 | 0.0000 | 0.0000 | 0.0024 | 0.0013 | -0.0010 |
| 9 | 0.0000 | 0.0000 | -0.0000 | 0.0013 | 0.0027 | 0.0014 |
| 10 | 0.0000 | 0.0000 | -0.0000 | -0.0010 | 0.0014 | 0.0024 |

Table 11. Simple effect estimates and 95% confidence intervals for simple effect contrasts of internal meta-analysis

| Contrast | Estimate | 95% *CI* |
| --- | --- | --- |
| 5 | 0.06 | -0.02 - 0.15 |
| 6 | 0.03 | -0.05 - 0.12 |
| 7 | -0.03 | -0.12 - 0.05 |
| 8 | 0.14 | 0.05 - 0.24 |
| 9 | 0.00 | -0.10 - 0.10 |
| 10 | -0.14 | -0.24 - -0.05 |
